# Habitat and Host Indicate Lineage Identity in *Colletotrichum gloeosporioides* s.l. from Wild and Agricultural Landscapes in North America

**DOI:** 10.1371/journal.pone.0062394

**Published:** 2013-05-06

**Authors:** Vinson P. Doyle, Peter V. Oudemans, Stephen A. Rehner, Amy Litt

**Affiliations:** 1 The New York Botanical Garden, Bronx, New York, United States of America; 2 The Graduate Center, City University of New York, New York, New York, United States of America; 3 Philip E. Marucci Center for Blueberry and Cranberry Research and Extension, Rutgers University, Chatsworth, New Jersey, United States of America; 4 Systematic Mycology and Microbiology Laboratory, United States Department of Agriculture-Agricultural Research Service, Beltsville, Maryland, United States of America; Soonchunhyang University, Republic of Korea

## Abstract

Understanding the factors that drive the evolution of pathogenic fungi is central to revealing the mechanisms of virulence and host preference, as well as developing effective disease control measures. Prerequisite to these pursuits is the accurate delimitation of species boundaries. *Colletotrichum gloeosporioides s.l.* is a species complex of plant pathogens and endophytic fungi for which reliable species recognition has only recently become possible through a multi-locus phylogenetic approach. By adopting an intensive regional sampling strategy encompassing multiple hosts within and beyond agricultural zones associated with cranberry (*Vaccinium macrocarpon* Aiton), we have integrated North America strains of *Colletotrichum gloeosporioides s.l.* from these habitats into a broader phylogenetic framework. We delimit species on the basis of genealogical concordance phylogenetic species recognition (GCPSR) and quantitatively assess the monophyly of delimited species at each of four nuclear loci and in the combined data set with the genealogical sorting index (*gsi*). Our analysis resolved two principal lineages within the species complex. Strains isolated from cranberry and sympatric host plants are distributed across both of these lineages and belong to seven distinct species or terminal clades. Strains isolated from *V. macrocarpon* in commercial cranberry beds belong to four species, three of which are described here as new. Another species, *C. rhexiae* Ellis & Everh., is epitypified. Intensive regional sampling has revealed a combination of factors, including the host species from which a strain has been isolated, the host organ of origin, and the habitat of the host species, as useful indicators of species identity in the sampled regions. We have identified three broadly distributed temperate species, *C. fructivorum*, *C. rhexiae*, and *C. nupharicola*, that could be useful for understanding the microevolutionary forces that may lead to species divergence in this important complex of endophytes and plant pathogens.

## Introduction

Delimiting species boundaries among fungi lays the groundwork for detailing the natural history and ecology of species and defines a robust framework from which further comparative studies can be designed (i.e. population genetics/genomics). This is also prerequisite to providing targeted and effective disease control measures and identifying specific pathogens against which plant breeders can focus their efforts in developing and selecting disease resistant cultivars. Strictly agro–centric studies of plant pathogens risk sampling too narrowly, overlooking important adjacent (parapatric) niches driving pathogen evolution. Extensive sampling, within, adjacent to, and beyond agricultural landscapes has the potential to provide a broader view of the natural history of pathogen species and offer insight into the evolution of differential traits among closely related lineages [1].

*Colletotrichum* Corda is among the most important and widespread genera of plant-associated fungi, causing disease and occurring as asymptomatic endophytes on aerial organs of a broad range of host plants [2]–[6]. *Colletotrichum gloeosporioides sensu lato* represents an aggregate of species frequently reported as a dominant endophyte of tropical herbaceous plants and is known as a field and post–harvest fruit pathogen of many economically important crops [7]–[11]. Morphological homoplasy and phenotypic plasticity have previously thwarted efforts to clearly define species boundaries within the species complex, necessary if we are to develop a greater understanding of the ecology and natural history of each lineage. The recent development of molecular markers suitable for resolving species limits and phylogenetic relationships within this species aggregate have been proposed and validated [12]–[16], making it possible to examine the role of geography, host preference/specificity, the nature of host–pathogen/host–endophyte associations, and other niche specialization attributes that may underlie species divergence [14]. Furthermore, the recent epitypification of *C. gloeosporioides* provides a much needed reference point for developing an improved taxonomic framework for the species complex [17].

The current study uses a phylogenetic approach to infer species boundaries among North American members of the *C. gloeosporioides* species complex, particularly those in association with the large American cranberry (*Vaccinium macrocarpon* Aiton) and sympatric plant species. *Colletotrichum gloeosporioides s.l.* has been reported as a leaf and fruit pathogen of cranberry since the late 1800s [18] and early 1900s [19] and has recently been observed to colonize stem tissue [20]. Contemporary studies of fruit pathogens have confirmed the importance of *C. gloeosporioides s.l.* in agricultural ecosystems throughout the cultivated range of *V. macrocarpon* [8], [21]–[23]. However, given the fact that *C. gloeosporioides s.l.* is an aggregate of species that can be difficult or impossible to distinguish morphologically, it is not clear that the strains isolated from cranberry are conspecific throughout the cultivated range of cranberry. In addition, genetic studies have focused on isolates solely from fruit and have not investigated diversity from alternate host organs or sympatric host species, despite evidence that these represent potential reservoirs of diversity for the species complex in cranberry agricultural areas [19], [24]–[27].

*Vaccinium macrocarpon* is one of North America’s few native, economically important crop species. Cranberry has undergone little selection from wild relatives and is cultivated in several regions alongside extant native populations [28]. It can also be found in sympatry with the closely related species *V. oxycoccos* L., which has been used for inter-specific hybridization in breeding programs but is not cultivated for agricultural production [29]. In addition, many of the cultivars used in past breeding regimes remain available for further experimentation. These characteristics provide cranberry breeders with excellent resources for meeting the needs of cranberry growers to improve fruit production and reduce pathogen pressure through breeding for disease resistance [30]. However, providing a refined systematic understanding of the pathogens that are the source of disease pressure will be necessary to help focus the efforts of plant breeders.

Host specificity/preference has historically been a focal criterion for species delimitation in *Colletotrichum* [7], [11]. Similarly, contemporary studies have indicated the presence of host specific lineages [13], [31]. However, host-fungus associations within *Colletotrichum* are variable. It is clear that multiple species are capable of infecting single hosts and, conversely, some *Colletotrichum* species are capable of infecting multiple host species [13], [14], [32]. In addition, there is indication that different plant organs may act as selective forces leading to organ specialization in *Colletotrichum* species [33]. While *C. gloeosporioides s.l.* is established as a pathogen of a variety of important crop species worldwide [8], [14], [15], [31], [34]–[38], this study represents a unique perspective into the evolution of the species complex by investigating the genetic diversity of *C. gloeosporioides s.l.* in a single crop species and its surrounding habitat within the host’s native range. The specific objectives of this study are to determine: (1) whether there are multiple sympatric lineages within the species complex that infect cranberry, 2) whether host preference/specificity is evident among sympatric lineages within the species complex, 3) whether host organ or nature of the fungus-host association is predictive of phylogenetic structure, and 4) whether we can identify lineages with broad geographical and/or host associations suitable for fine-scale landscape genetic analysis. We sampled horizontally across five sympatric host species in wild and commercial cranberry bogs in order to address questions related to host specificity, and sampled vertically among different plant organs within *V. macrocarpon* to target questions related to organ specialization (Table 1). Utilizing the recent development of molecular phylogenetic markers useful for distinguishing lineages within the species complex [12]–[15] and isolates from five major cranberry agricultural areas, wild cranberry bogs, fruit and stem of *V. macrocarpon*, and from five sympatric host species, we assess lineage diversity among isolates from cranberry and surrounding habitats.

**Table 1:** *Colletotrichum* strains included in the four marker (D4G) phylogenetic analysis.

Strain	Locality	Host	Organ	Species
Coll57	New Jersey	*Vaccinium macrocarpon*	Stem	*C. aff. acutatum*
Coll60	New Jersey	*V. macrocarpon*	Fruit	*C. aff. acutatum*
CBS124	New Jersey	*V. macrocarpon*	NA	*C. fructivorum*
Coll1002 = CBS133128	British Columbia	*V. macrocarpon*	Fruit	*C. fructivorum*
Coll1004	British Columbia	*V. macrocarpon*	Fruit	*C. fructivorum*
Coll1062	Massachussets	*V. macrocarpon*	Fruit	*C. fructivorum*
Coll1081 = CBS133121	Massachussets	*V. macrocarpon*	Fruit	*C. fructivorum*
Coll1092* = CBS133135	New Jersey	*V. macrocarpon*	Stem	*C. fructivorum*
Coll116	Massachussets	*V. macrocarpon*	Fruit	*C. fructivorum*
Coll1164 = CBS133126	Wisconsin	*V. macrocarpon*	Fruit	*C. fructivorum*
Coll1178	Wisconsin	*V. macrocarpon*	Fruit	*C. fructivorum*
Coll1190	Wisconsin	*V. macrocarpon*	Fruit	*C. fructivorum*
Coll1216	Wisconsin	*V. macrocarpon*	Fruit	*C. fructivorum*
**Coll1414 = CBS133125**	New Jersey	*V. macrocarpon*	Fruit	*C. fructivorum*
Coll445 = CBS133127	New Jersey	*V. macrocarpon*	Fruit	*C. fructivorum*
Coll21 = CBS133133	New Jersey	*V. macrocarpon*	Fruit	*C. fructivorum*
Coll864 = CBS133130	New Jersey	*Rhexia virginica*	Stem	*C. fructivorum*
Coll873	Delaware	*V. macrocarpon*	Fruit	*C. fructivorum*
Coll886 = CBS133124	Pennsylvania	*Vaccinium oxycoccos*	Fruit	*C. fructivorum*
**Coll1026 = CBS133134**	Delaware	*Rhexia virginica*	Stem	*C. rhexiae*
Coll1034 = CBS133136	Delaware	*V. macrocarpon*	Fruit	*C. rhexiae*
Coll1041	Delaware	*V. macrocarpon*	Fruit	*C. rhexiae*
Coll877* = CBS133132	Delaware	*V. macrocarpon*	Fruit	*C. rhexiae*
Coll1038	Delaware	*R. virginica*	Stem	*C. rhexiae*
Coll1306	New Jersey	*R. virginica*	Stem	*C. rhexiae*
Coll952 = CBS133131	New Jersey	*R. virginica*	Stem	*C. rhexiae*
Coll1470 = CBS133129	Maryland	*R. virginica*	Leaf	*C. rhexiae*
GJS08214	Cameroon	*Coffea arabica*	Fruit	*C. kahawae*
GJS08216	Cameroon	*C. arabica*	Fruit	*C. kahawae*
GJS08211	Cameroon	*C. arabica*	Fruit	*C. kahawae*
**IMI319418**	Kenya	*C. arabica*	Fruit	*C. kahawae*
Coll1103* = CBS133120	New Jersey	*V. macrocarpon*	Stem	*C. temperatum*
**Coll883 = CBS133122**	New York	*V. macrocarpon*	Fruit	*C. temperatum*
Coll11*	Florida	*Persea americana*	Leaf	*C. tropicale*
Coll918	Puerto Rico	*Terpsichore taxifolia*	Sporangium	*C. tropicale*
GJS0842	Panama	*Annona muricata*	Leaf	*C. tropicale*
4861	Panama	*T. cacao*	Leaf	*C. tropicale*
**5101** *	Panama	*T. cacao*	Leaf	*C. tropicale*
8401*	Panama	*T. cacao*	Leaf	*C. tropicale*
E1164*	Panama	*Trichilia tuberculata*	Leaf	*C. tropicale*
E2303	Panama	*Virola surinamensis*	Leaf	*C. tropicale*
E406	Panama	*Pentagonia macrophylla*	Leaf	*C. tropicale*
Q633*	Panama	*Cordia alliodora*	Leaf	*C. tropicale*
7423*	Panama	*Theobroma cacao*	Leaf	*C. tropicale*
Coll126 = CBS133123	New Jersey	*V. macrocarpon*	Stem	*C. melanocaulon*
**Coll131 = CBS133251**	New Jersey	*V. macrocarpon*	Stem	*C. melanocaulon*
Coll6	Florida	*Mangifera indica*	Leaf	*C. siamense*
1092	Panama	*Theobroma cacao*	Leaf	*C. siamense*
CollNC67	North Carolina	NA	NA	*C. siamense*
Coll54	New Jersey	*Prunus persica*	Fruit	*C. sp. indet. B*
CollNC60	North Carolina	NA	?	*C. sp. indet. B*
GJS0852	Panama	*T. cacao*	Leaf	*C. sp. indet. A*
7767	Panama	*T. cacao*	Leaf	*C. sp. indet. A*
GJS0857	Panama	*P. americana*	Fruit	ND
Coll38	Florida	*M. indica*	Fruit	*C. asianum*
GJS08144	Panama	*M. indica*	Fruit	*C. asianum*
GJS08147	Panama	*M. indica*	Fruit	*C. asianum*
Coll1126*	New Jersey	*V. macrocarpon*	Stem	*C. fructicola*
Coll996	New Jersey	*R. virginica*	Stem	*C. fructicola*
CollP1	North Carolina	*V. corymbosum*	Leaf	*C. fructicola*
1087*	Panama	*T. cacao*	Leaf	*C. fructicola*
3589*	Panama	*T. cacao*	Leaf	*C. fructicola*
3679*	Panama	*T. cacao*	Leaf	*C. fructicola*
**7574**	Panama	*T. cacao*	Leaf	*C. fructicola*
E886	Panama	*Tetragastris panamensis*	Leaf	*C. fructicola*
Coll878	New Jersey	*Nuphar lutea*	Leaf	*C. nupharicola*
**cbs470**	Washington	*N. lutea ssp. polysepala*	Leaf	*C. nupharicola*
Coll922	Washington	*N. lutea*	Leaf	*C. nupharicola*
cbs472	Rhode Island	*Nymphaea odorata*	Leaf	*C. nupharicola*
Coll919	Puerto Rico	*T. taxifolia*	Leaf seta	*“C. ignotum 2″*
8395	Panama	*T. cacao*	Leaf	*“C. ignotum 2″*
E183	Panama	*Genipa americana*	Leaf	*“C. ignotum 2″*
Coll940	Oklahoma	*Juglans nigra*	Leaf	ND
Coll887	W. Virginia	*V. oxycoccos*	Fruit	*C. sp. indet. C*
Coll920	New Jersey	*Chamaecyparis thyoides*	Stem	*C. sp. indet. C*
Coll20*	Mexico	*Solanum sp.*	stem	*C. gloeosporioides*
IMI356878	Italy	*Citrus sinsensis*	Fruit	*C. gloeosporioides*
Coll914	Mexico	*Solanum sp.*	Leaf	*C. gloeosporioides*
GJS0848	Panama	*T. cacao*	Fruit	*C. theobromicola*
GJS0843	Panama	*T. cacao*	Leaf	*C. theobromicola*
GJS0850	Panama	*T. cacao*	Leaf	*C. theobromicola*
3386	Panama	*T. cacao*	Leaf	*C. sp. indet. D*
4801	Panama	*T. cacao*	Leaf	*C. sp. indet. D*
4766	Panama	*T. cacao*	Leaf	*C. sp. indet. D*

Accession numbers for cultures deposited at Centraalbureau voor Schimmelcultures (CBS) are included for relevant strains.

* Endophyte isolates.

ND – not designated.

NA – not available.

Type strains are in bold-faced type.

## Methods

### Ethics Statement

All necessary permits were obtained for the described field studies from the Delaware Division of Parks and Recreation, the National Forest Service, and The Nature Conservancy. All other samples collected in this study were on private land and did not require permits. There were no endangered or protected species collected for this study.

### Fungal Isolation and Culturing

*Colletotrichum* was isolated from symptomatic and asymptomatic tissue of several host species in North America with a focus on species sympatric with *Vaccinium macrocarpon* in wild and agricultural habitats. Sympatric host plant species from which *C. gloeosporioides s.l.* was isolated include *Vaccinium oxycoccos, Rhexia virginica* L., *Chamaecyparis thyoides* (L.) Britton, Sterns, & Poggenb., and *Nuphar lutea* (L.) Sm. (Table 1). *Colletotrichum gloeosporioides s.l.* was isolated from symptomatic and asymptomatic stem and fruit of *V. macrocarpon* (Table 1) in both wild and agricultural habitats. Both symptomatic and asymptomatic tissue was surface sterilized in 10% bleach (final concentration 0.6125% sodium hypochlorite) between 1.5 minutes and 5 minutes, depending on the permeability of the tissue, and plated on V8 juice agar, 2% malt extract agar (MEA: BD Diagnostics, Franklin Lakes, NJ, USA) or corn meal agar (CMA: BD Diagnostics), with the exception of *Nuphar lutea. Nuphar lutea* was incubated in a humid chamber under ambient light and conidia were transferred from anthracnose lesions to CMA and potato dextrose agar (PDA: BD Diagnostics) for purification. Isolates were characterized as endophytes if they were isolated from asymptomatic tissue after surface sterilization; all other isolates were considered pathogenic. Strains morphologically similar to *Colletotrichum gloeosporioides s.l.*, based on an assortment of characters including growth rate, colony color, hyphal morphology and/or conidial shape and size, were isolated by transferring conidia or hyphal tips to sterile media and preserved on CMA slants stored at 6°C, stored in 1.5 mL microcentrifuge tubes at 6°C, and in 10% glycerol at −80°C. Types, epitypes, and representative cultures were deposited at the Centraalbureau voor Schimmelcultures (CBS) with corresponding dried cultures deposited as vouchers at the U.S. National Fungus Collections (BPI).

### DNA Extraction and PCR Amplification

Isolates were grown on potato dextrose broth (Difco) for 5–7 days before mycelium was harvested, blotted dry on sterile paper towels and dehydrated for 6–10 hours in a vacuum centrifuge on low heat. Approximately 20 mg of dried tissue was used for DNA extraction using the DNeasy Plant MiniKit (Qiagen Inc., Valencia, CA, USA) or a standard phenol-chloroform extraction method after tissue homogenization in the FastPrep FP120 (MP Biomedicals, LLC., Solon, OH, USA).

PCR amplification reactions of nuclear ribosomal internal transcribed spacer (nrITS), *beta-tubulin* (*tub2*), DNA lyase (*apn2*), and an intergenic spacer between the 3′ end of the DNA lyase and the mating type locus MAT1-2 (*apn2/matIGS*) were performed in 25 µl reactions containing 9.3 µL of autoclaved ion-exchanged water, 2.5 µL of dNTP mixture (2.5 mM stock of each dNTP), 2.5 µL of bovine serum albumin (BSA; 0.25 µg µL−1 stock), 2.5 µL of buffer [200 mm Tris pH 8.8, 100 mM KCl, 100 mM (NH_4_)_2_SO_4_, 20 mm MgSO_4_·7H_2_O, 1% (v/v) Triton X-100, 50% (w/v) sucrose, 0.25% (w/v) cresol red], 5 µL betaine (1.2 M stock), 1 µL of each primer (0.67 µM final concentration), 0.2 µL of *Taq* polymerase (GenScript USA Inc., Piscataway, NJ, USA) and 1 µL (approximately 20–50 ng) of genomic DNA. The nrITS region was amplified and sequenced using primers ITS5 and ITS4 [39], *tub2* with primers T1 and T224 [40], *apn2* with CgDL_R1 and ColDL_F3 [14], and *apn2mat/IGS* using primers CgDL_F6 and CgMAT1_F2 [14].

PCR reactions were run on an Eppendorf Mastercycler pro S with the following cycling parameters: nrITS–initial denaturing for 2 min at 94°C, followed by 38 cycles of 94°C for 1 min, 55°C for 30 s, and 72°C for 45 s, followed by a final extension at 72°C for 5 min; *tub2*– initial denaturing for 2 min at 94°C, followed by 30 cycles of 94°C for 35 s, 52°C for 55 s, and 72°C for 2 min, followed by a final extension at 72°C for 5 min; *apn2/matIGS*–initial denaturing for 5 min at 95°C, followed by 10 cycles of 95°C for 30 s, 62°C (decreasing by 1°C each cycle) for 30 s, and 72°C for 1 min, followed by 35 cycles of 95°C for 30 s, 52°C for 30 s, and 72°C for 1 min, followed by a final extension at 72°C for 10 min. PCR products were purified and sequenced at the High Throughput Genomics Unit, Department of Genome Sciences, University of Washington using ABI 3730×l sequencers.

### Contig Assembly, Sequence Editing, and Phylogenetic Inference

Sequences were automatically assembled into contigs, and edited manually in Sequencher version 4.9 (GeneCodes Corp., Ann Arbor, Michigan). Alignments were carried out with the online version of the sequence alignment program MAFFT version 6 [41], [42] using the iterative refinement option G-INS-i for each locus independently. GenBank accession numbers for sequences generated for this study are provided in Table S1.

Rojas et al. [14] sampled New World isolates of *C. gloeosporioides s.l.* representing several lineages within the species complex and demonstrated the utility of a combined analysis of nrITS, *btub*, *apn2*, and *apn2/matIGS* to resolve closely related lineages. Representative sequences from their study of all four markers were combined with sequences generated here to meet the aforementioned objectives. Independent gene trees were inferred under the parsimony (MP) optimality criterion in TNT [43] and maximum likelihood (ML) criterion in RAxML–HPC2 (7.2.8) implemented on the CIPRES Science Gateway portal [44]–[46]. Phylogenetic estimates of the concatenated matrix were inferred under parsimony in TNT, maximum likelihood in RAxML–HPC2 (implemented on the CIPRES cluster), and Bayesian inference in MrBayes v3.1.2 [47], [48]. Phylogenetic analyses in TNT were carried out with parsimony ratchet tree searches (one thousand random addition sequence replicates while holding 20 trees per replicate) and TBR branch swapping. Statistical support for the inferred nodes was determined with parsimony bootstrapping on 1000 pseudoreplicate datasets. Rapid bootstrapping in RAxML was carried out implementing the GTRCAT model and the ML tree search under the GTRGAMMA model (-m GTRCAT -x -f a). The best-fit models for Bayesian analyses were selected with MrModeltest 2.3 [49].

Independent analyses of nrITS and partial *beta-tubulin* were rooted in TNT and RAxML to *Coll57* and *Coll60*, strains of *Colletotrichum aff. acutatum* Simmonds, a species complex related to *Colletotrichum gloeosporioides s.l* [50]–[52]. Strains *Coll57* and *Coll60* were identified based on morphological similarity and NCBI BLAST similarity searches of nrITS and partial *beta-tubulin* (nrITS: *Glomerella fioriniae –*GenBank accession JN121193, e-value 0.0; *btub*: *Glomerella acutata* – GenBank accession AB273716, e-value 0.0). Analyses of *apn2* and *apn2/matIGS* were rooted in TNT and RAxML to strain *4766*, which exploratory nrITS and *tub2* analyses and a previous study of the group [14] indicated is a suitable outgroup for inferring phylogenetic relationships within *C. gloeosporioides s.l*. Statistical support for the inferred nodes was determined in RAxML–HPC2 by bootstrapping with the number of replicates for the independent gene trees determined by implementation of the extended majority rule (–N autoMRE) convergence criterion implemented in the CIPRES portal and the combined dataset with 1000 pseudoreplicates (–N 1000). Node frequencies for both parsimony and maximum likelihood bootstrap analyses were calculated with the SumTrees 3.1.0 program using the DendroPy Phylogenetic Computing Library version 3.7.1 [53].

The best-fit models implemented in MrBayes for each of nrITS, *tub2*, *apn2* and *apn2/matIGS* respectively, were as follows: SYM+Γ, GTR+I, GTR+Γ, HKY+ Γ. Four parallel runs were conducted with one cold and three heated Markov chains per run for 10,000,000 generations sampling every 1,000 generations, with each of these models applied independently to each locus in MrBayes. Convergence of parameter estimates was monitored in Tracer v. 1.5 [54] and posterior probabilities calculated from the sampled trees after discarding the first 25% as burn-in. To assess convergence to a global optimum in both ML and Bayesian tree searches of the combined dataset, the log-likelihood scores (−lnL) for the best tree from the maximum likelihood and Bayesian tree searches were calculated in RAxML [45].

In order to estimate the primary concordance tree from the multilocus dataset we used Bayesian concordance analysis (BCA) implemented in BUCKy v. 1.4.0 [55], [56]. BUCKy integrates over gene tree uncertainty to estimate the proportion of sequenced markers that support each clade and constructs the tree that reflects the dominant vertical phylogenetic signal (primary concordance tree) [56]. We limited our analysis with BUCKy to a reduced dataset of 55 unique haplotypes (sequences with missing data were considered unique). The posterior distribution of single gene trees was inferred in MrBayes using the same model parameters for each partition as in the concatenated dataset. The primary concordance tree and concordance factors (CF) were estimated across a range of prior values for the discordance parameter (α) of 0.1, 1, 10, and 100. The discordance parameter (α) represents the prior probability distribution that all genes share the same tree, where α = 0 indicates all genes share the same tree while α = ∞ indicates all genes have a distinct set of trees [55]. All analyses were initially run with two Markov chain Monte Carlo search chains (MCMC) of 100,000 generations after a burn-in of 10,000 generations. All runs converged on the same primary concordance tree with identical concordance factors. The final analysis was run with α = 1 and two MCMC chains of 1,000,000 generations following a burn-in of 100,000 generations (-a 1 -c 2 -n 1000000).

Several species within the *C. gloeosporioides* species complex have been published since the epitypification of *C. gloeosporioides* [14], [17], [57], [58] and a comprehensive phylogenetic analysis of the species complex, including the description of new species and subspecies, was recently published [16]. Despite agreement regarding the necessity of multilocus genetic data to resolve taxonomic problems within the species complex and the recognition that the phylogenetic resolution provided by the most commonly used markers is significantly lacking, a common set of markers has not yet been adopted among independent research groups [14], [15], [17], [57]–[59]. Nonetheless, there is some overlap in the markers sequenced for recently described species. In order that isolates from this study could be placed in the broader context of the species complex and to evaluate the distinctiveness of the newly described species presented here (see ‘Species assignments’ below), we analyzed sequence data from representative isolates and type strains available in GenBank. In addition to the complete four-marker dataset described above, hereafter referred to as D4G, two additional datasets were constructed with the available sequence data from nrITS, partial *tub2*, and *apn2/matIGS.* The first dataset, hereafter referred to as D3G, included all isolates from the D4G dataset with the addition of sequence data from nine isolates, included in a recent study by Silva et al. (2012), representing three additional species and one subspecies. The combined data set included 2,134 nucleotides (nrITS: 557; *tub2*∶690; *apn2mat/IGS*: 887) for 91 isolates with no missing markers for any terminal. The second dataset, hereafter referred to as D3G^+^, included the addition of nrITS and partial *tub2* sequence data for thirty additional strains, included in a comprehensive phylogenetic study of the species complex by Weir et al. (2012), representing sixteen species and one *forma speciales* with data for the *apn2/matIGS* marker coded as missing for these additional strains in an alignment of 2,148 nucleotides. Phylogenetic analyses of these concatenated matrices were conducted with RAxML–HPC2 and MrBayes v3.1.2 as previously described. The best-fit models determined in MrModeltest 2.3 for each locus in the expanded datasets were as follows: nrITS – GTR+Γ; *tub2–* GTR+Γ; *apn2/matIGS* – HKY+ Γ. GenBank accession numbers from previously published studies included in analyses presented here are provided in Table S2 and all multiple sequence alignments are available from the authors upon request. The presentation of results from the analysis of D3G^+^ is restricted to Text S1 for the sake of brevity and clarity.

### Species Assignments

In order to delimit novel species, we applied the criteria of genealogical concordance phylogenetic species recognition (GCPSR) [60], [61] to phylogenetic estimates from the D4G dataset. Briefly, following Dettman et al. [60], novel species were recognized if they satisfied one of two criteria: genealogical concordance or genealogical non-discordance. Clades were genealogically concordant if they were present in three of the four single gene trees and genealogically non-discordant if they were strongly supported (MP≥75%; ML≥70%) in a single gene and not contradicted at or above this level of support in any other single gene tree. In addition, novel species were recognized if resolved with strong support (PP≥.95; ML≥70%; MP≥75%) in all analyses for the combined dataset of nrITS, *tub2*, *apn2*, and *apn2/matIGS* and were not nested within clades containing the type of any previously described species in any of the combined analyses. All isolates were assigned to a species with the exception of *GJS0857* and *Coll940,* which remain singletons.

### Quantitative Measures of Genealogical Sorting

The genealogical sorting index is used here to represent a quantitative assessment of the monophyly or genealogical exclusivity of a group of commonly labeled terminals in a set of trees. This quantitative measure enables the comparison of individual markers to assess their ability to differentiate lineages or species. In order to determine the degree of exclusive ancestry of each of the well–supported lineages inferred from the concatenated dataset of nrITS, *btub*, *apn2*, and *apn2mat/IGS* (D4G) across the independent gene trees, the bootstrap trees obtained from the parsimony bootstrap analyses for each independent gene and the concatenated dataset were used to calculate the genealogical sorting index (*gsi*) for each lineage [62]. The *gsi* was calculated for lineages of the *C. gloeosporioides* species aggregate using the R (2.13.0) package genealogicalSorting version 0.91 [63], [64] after removing outgroup strains (*Coll57, Coll60, 4766, 4801, 4766*). The genealogical sorting index (*gsi*) reaches a maximum when a commonly labeled group (species or terminal lineage) reaches monophyly in a tree or set of trees and a minimum when all nodes on the tree are required to unite a group. P-values represent the probability that the calculated *gsi* from the inferred tree or trees would be observed by chance. The ensemble genealogical sorting index (*gsi_T_*) is the sum of the *gsi* values for each topology weighted by the probability of a given topology based on the proportion of trees in the sample where it is represented. P-values are calculated by permutation of group labels on the terminals of a tree and determining the frequency distribution from the recalculated *gsi* value. P-values are estimated from 1,000 permutations of each dataset and represent the probability of observing *gsi* values ≥ to the reported *gsi* values by chance under the null hypothesis that labeled groups are of mixed ancestry.

### Morphological Studies

Morphological observations were made from strains cultured on potato dextrose agar (PDA), corn meal agar (CMA), V8 agar, and Synthetischer nährstoffarmer agar (SNA) [65] incubated at 22–25°C. Growth rates were determined from cultures grown in darkness at 25°C on PDA. Cultures were first grown on CMA and 10 mm plugs were taken from the expanding margin and transferred to 20 mL of PDA in 100×15 mm Petri dishes. Each strain was plated to three replicate plates. Three radial measurements were taken from the edge of the plug to the margin of the colony every 24–48 hours over the course of 5 days by marking the bottom of the plate at the margin of the colony using a stereomicroscope equipped with stage lighting, resulting in 9 radial measurements per strain. Each plate was then photographed and the distance between markings was measured.

Microscopic observations of conidia, phialides, and ascospores were made from specimens mounted in water. Hyphal appressoria were observed using slide cultures; a 10 mm^2^ block of CMA was placed on a CMA plate, each of the four corners of the block was inoculated, covered with a sterile coverslip, and incubated at 25°C. Microscopic observation of perithecial development on cranberry fruit was made by surface sterilizing symptomatic field collected fruit, cutting in half transversely, placing face down on V8 agar, and incubating at room temperature (22°C) for approximately 3 weeks. The fruit was removed from the plate after perithecial development and fixed in FAA (3.7% formaldehyde, 5% glacial acetic acid, and 50% ethanol) before dehydration in an alcohol-xylene dehydration series and embedded in Paraplast X-TRA (Leica Microsystems, Buffalo Grove, IL, USA). The fruit was sectioned at 8 µm and stained with acid fuchsin and Cotton blue in lactic acid and mounted in Permount (Fisher Scientific, Pittsburgh, PA, USA).

Microscopic images were made with a Nikon DXM1200C digital camera attached to a Zeiss Axioplan compound microscope or with a Nikon SMZ1500 stereoscope equipped with a Nikon DXM1200F digital camera using Nikon ACT-1 software. All measurements were made using ImageJ 1.44p software [66], [67] and summary statistics calculated in R version 2.13.0. Growth rates are reported giving the minimum, average, maximum [(minimum-) average (-maximum)] and the standard deviation in millimeters per day. All other measurements additionally include the 1^st^ quartile and the 3^rd^ quartile with the following notation: (minimum-) 1^st^ quartile – average –3^rd^ quartile (-maximum).

### Nomenclature

The electronic version of this article in Portable Document Format (PDF) in a work with an ISSN or ISBN will represent a published work according to the International Code of Nomenclature for algae, fungi, and plants, and hence the new names contained in the electronic publication of a PLOS ONE article are effectively published under that Code from the electronic edition alone, so there is no longer any need to provide printed copies.

In addition, new names contained in this work have been submitted to MycoBank from where they will be made available to the Global Names Index. The unique MycoBank number can be resolved and the associated information viewed through any standard web browser by appending the MycoBank number contained in this publication to the prefix http://www.mycobank.org/MB. The online version of this work is archived and available from the following digital repositories: PubMed Central; LOCKSS.

## Results

### Individual Gene Trees

In order to test the hypothesis that multiple sympatric lineages within the *C. gloeosporioides* species complex infect cranberry in the field, North American isolates need to be placed in a broader phylogenetic context. Therefore, sequence data generated in this study were combined with an earlier study of isolates of *C. gloeosporioides s.l.* from the New World tropics [14]. Outgroup sampling was expanded from the aforementioned study to include two isolates of *C. aff. acutatum*. Individual locus data were analyzed separately to assess the topological congruence among datasets and the utility of each to resolve terminal lineages with robust statistical support for sister group relationships within the *C. gloeosporioides* species complex. Strain data are summarized in Table 1 and character and tree statistic data are summarized in Table 2.

**Table 2:** Tree statistics.

Locus	# Taxa	# Chars	% PIC	#MPTs	MPT length	CI	RI	ML -ln L	BI -ln L
nrITS	84	570	13.3	28	93	84	95	−1324.92	NA
*tub2*	84	900	31.2	12	403	87	97	−3389.23	NA
*apn2*	82	756	27.3	14	339	79	96	−3024.04	NA
*apn2/MAT12IGS*	82	893	52.1	1	723	83	97	−4531.11	NA
Combined	84	3119	33	1	1585	82	96	−12981.97	−12981.97

# Taxa: number of taxa included in the analysis.

# Chars: number of characters per locus. %PIC: Percent of parsimony informative characters.

# MPTs: number of most parsimonious trees. MPT: length of the most parsimonious trees. CI: ensemble consistency index. RI: ensemble retention index. ML –ln L: ML score of best tree calculated in RAxML under the GTRGAMMA model. BI –ln L: ML score of best tree from 4 runs of 10,000,000 generations inferred in MrBayes calculated in RAxML with GTRGAMMA model.

Sequence data from nrITS has been widely used in fungal phylogenetic studies and has been proposed as a barcode locus for fungi [68]. However, our analysis indicates it neither provides adequate resolution for reliable species assignment, nor does it reliably assess phylogenetic relationships within the *C. gloeosporioides* species complex, as has been reported in previous studies of *Colletotrichum* [14], [23], [69]. Despite the low phylogenetic resolution inferred from nrITS data, six nodes within the species complex were supported in more than 75% of the parsimony bootstrapped datasets (Figure S1) and eight nodes in more than 70% of the maximum likelihood bootstrapped datasets (Figure S5). In addition, *C. sp. indet. D* (*4766*, *3386*, *4801*) was determined to be closely related but peripheral to the *C. gloeosporioides* species complex, indicating this is a suitable outgroup, as previously suggested by Rojas et al. [14].

Phylogenetic analysis of partial *tub2* sequence data largely supported the inferences made from nrITS data, but provided further resolution within the species complex, recovering 20 well-supported nodes in both MP and ML analyses (Figures S2 and S6). Isolates from cranberry (*V. macrocarpon* and *V. oxycoccos*) were distributed among 5 clades, including lineages originally described from tropical regions.

Phylogenetic analysis of the *apn2* locus provided greater resolution for terminal lineages than that achieved with either *tub2* or nrITS. Bootstrap analyses provided significant statistical support for 23 nodes and 20 nodes for MP (Figure S3) and ML (Figure S7) analyses, respectively, with six clades in both analyses containing isolates from *V. macrocarpon* and *V. oxycoccos*. While support for terminal lineages from *apn2* sequence data was greater than that from partial *tub2*, support for sister group relationships was not as robust.

A similar topology was recovered from the analysis of nucleotide data from *apn2/matIGS*, the intergenic spacer bridging the *apn2* and mating-type locus, with respect to the nrITS, *tub2*, and *apn2* gene trees while providing greater resolution than the other three datasets. Bootstrap analyses of *apn2/matIGS* recovered strong branch support for 25 and 29 nodes for MP (Figure S4) and ML (Figure S8), respectively. In agreement with the *apn2* analysis, six lineages include isolates from *V. macrocarpon* and *V. oxycoccos*. In addition to increased resolution of terminal clades within the species complex, compared with *apn2*, support for sister group relationships within the species complex is stronger than inferences based on partial *tub2*.

While statistical node support based on bootstrap resampling was somewhat inconsistent among ML and MP analyses, both sets of analyses were concordant, with differing levels of resolution. Similarly, the resolution of terminal clades among independent gene trees is consistent with a few exceptions. One strain, isolated as a leaf endophyte of *Persea americana* (*Coll11*), was relegated with strong support to a terminal clade that includes isolates designated as *Colletotrichum siamense* based on analysis of nrITS, but this isolate is strongly supported in both the partial *tub2* and *apn2/matIGS* tree as belonging to a lineage that includes isolates of *C. tropicale.* The resolution of this isolate in the *apn2* analysis was ambiguous. The phylogenetic placement of another strain, *Coll940*, is inconsistent among gene trees. While *Coll940* is resolved as sister to a group that includes *C. fructicola,* “*C.* ignotum 2″, *C. nupharicola* and *C. sp. indet. C* in the *tub2* (ML) and *apn2* (MP) analyses, its placement is switched with *C. sp. indet. C* in the *apn2/matIGS* analyses.

### Phylogenetic Analysis of the Combined Datasets

**Figure 1:**
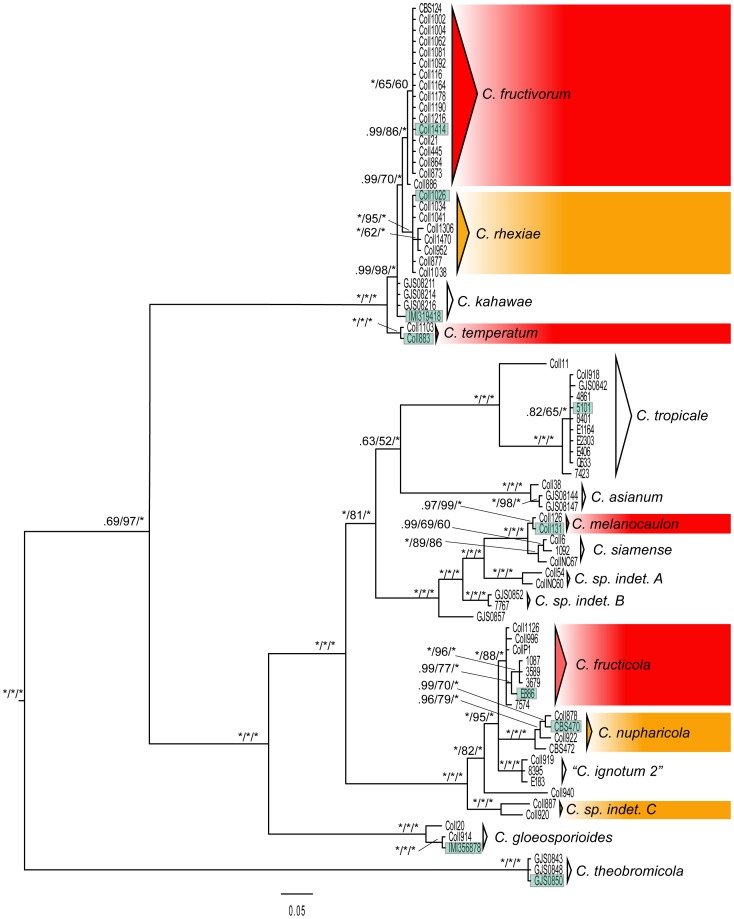
Bayesian majority rule consensus tree with support values (PP/ML-BS/MP-BS) for the combined analysis of nrITS, *btub*, *apn2*, and *apn2mat/IGS*. Outgroup taxa (*Coll57, Coll60, 3386, 4801, 4766*) have been trimmed from the phylogram. Support values of 1.0 or 100% are represented with “*”. Terminals enclosed in shaded boxes represent sequence data from ex-type strains. Clades enclosed in red boxes contain isolates from *Vaccinium macrocarpon* in cultivated cranberry beds in North America. Clades enclosed in orange boxes contain isolates from host species sympatric with cultivated *V. macrocarpon*.

In order to infer organismal phylogenetic relationships and make species assignments, we relied on the combined dataset of 3,119 nucleotide characters from four nuclear markers and eighty-four terminals (D4G). The Bayesian consensus tree is presented in Figure 1 with node posterior probability and bootstrap proportion values from Bayesian, parsimony, and maximum likelihood analyses. The outgroup taxa, *C. aff. acutatum* and *C. sp. indet. D* have been trimmed from Figure 1 due to long branches between the species complex and the outgroup terminals. The relationships among clades in each of the three analyses converged on a topology, with varying levels of node support, identical to the Bayesian analysis presented in Figure 1. The log-likelihood of the tree with the best score (highest log-likelihood value) from the set of four Bayesian runs as calculated in RAxML under the GTRGAMMA model (−1298.97) was identical to the tree inferred under the maximum-likelihood criterion.

Thirty-six nodes were inferred with strong bootstrap and posterior probability support in the combined analysis of nrITS, *tub2*, *apn2*, and *apn2mat/IGS*. The multilocus analyses corroborated the majority of the single gene trees in recovering *C. theobromicola* as sister to two principal lineages within the species complex. The first lineage includes two previously described species, *C. kahawae* and *C. rhexiae,* and two newly described species, *C. temperatum* and *C. fructivorum.* The second lineage includes six previously described species, *C. tropicale, C. asianum, C. siamense, C. fructicola, C. nupharicola,* and *C. gloeosporioides*, one new species, *C. melanocaulon* and four undescribed sublineages. Added taxon sampling suggests the original circumscription of *C. ignotum* (recently placed in synonymy with *C. fructicola* by Weir et al. [16]) to include isolates in the lineage labeled “*C. ignotum 2″* in Figure 1 was too broad, also discussed in Rojas et al. [14].

**Figure 2:**
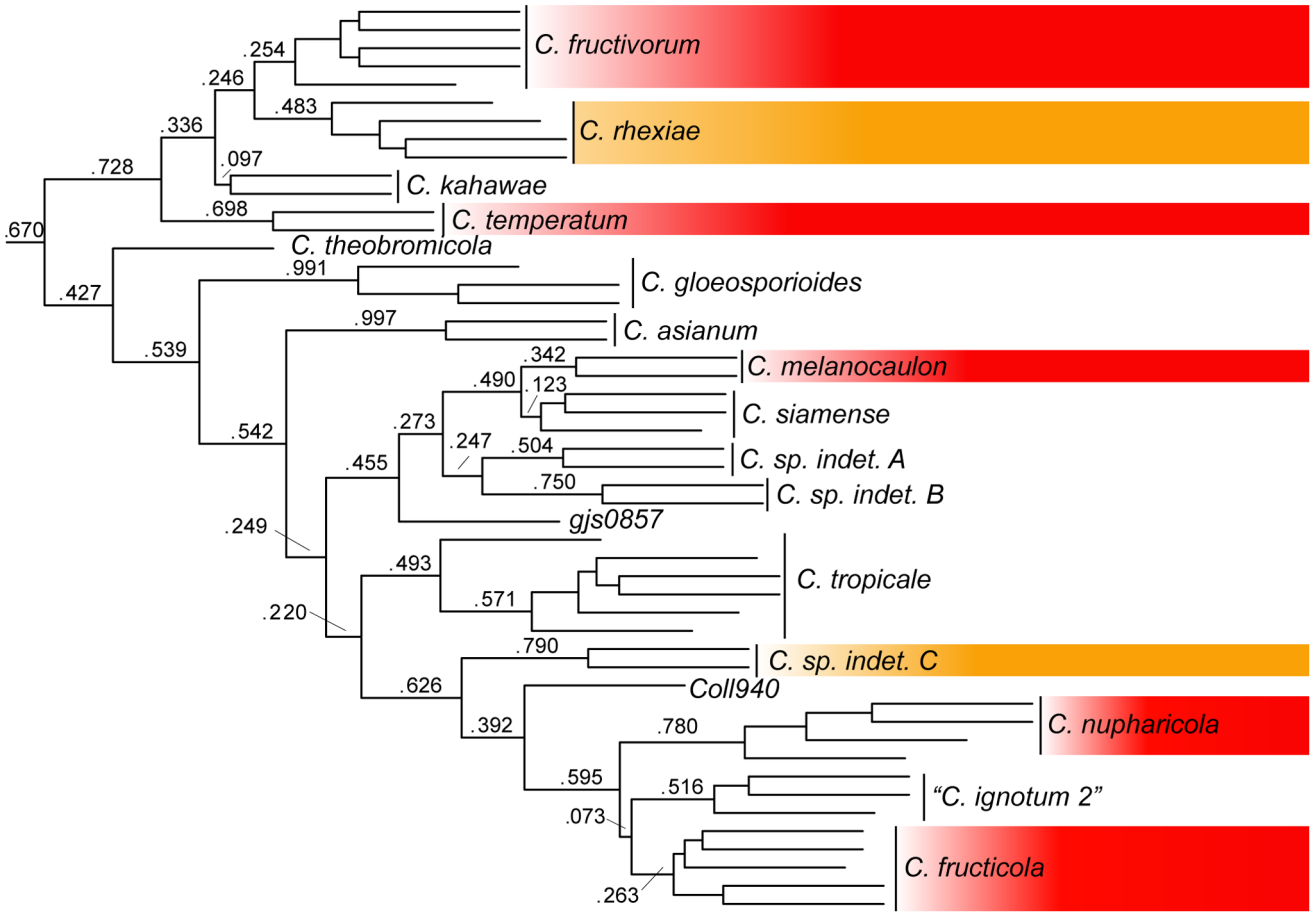
Primary concordance tree resulting from the Bayesian concordance analysis of the nrITS, *btub*, *apn2*, and *apn2mat/IGS* with concordance factors for nodes above the species level. Clades enclosed in red boxes contain isolates from *Vaccinium macrocarpon* in cultivated cranberry beds in North America. Clades enclosed in orange boxes contain isolates from host species sympatric with cultivated *V. macrocarpon*.

The primary concordance (PC) tree estimated with Bayesian concordance analysis from the four nuclear markers remained unchanged regardless of the value of the discordance parameter (α). The PC tree (Figure 2) is consistent with the assignment of individual isolates to terminal lineages on the basis of the concatenated analysis. Concordance factors are reported for all nodes above the species level. The low concordance factors (below 0.5) for several species, including *C*. *fructicola*, *C. asianum, C. siamense, C. kahawae*, *C. rhexiae,* and *C. fructivorum* appear to be due to the lack of resolution provided by individual markers rather than topological discordance. Similar to the analysis of the combined data, two principal lineages were resolved in the PC tree. However, relationships among a few species within these two lineages are distinctive from the combined analysis. For example, *Colletotrichum theobromicola* is placed within the principal lineage that includes *C. gloeosporioides,* and *C. asianum* is no longer sister to *C. tropicale.*

**Figure 3:**
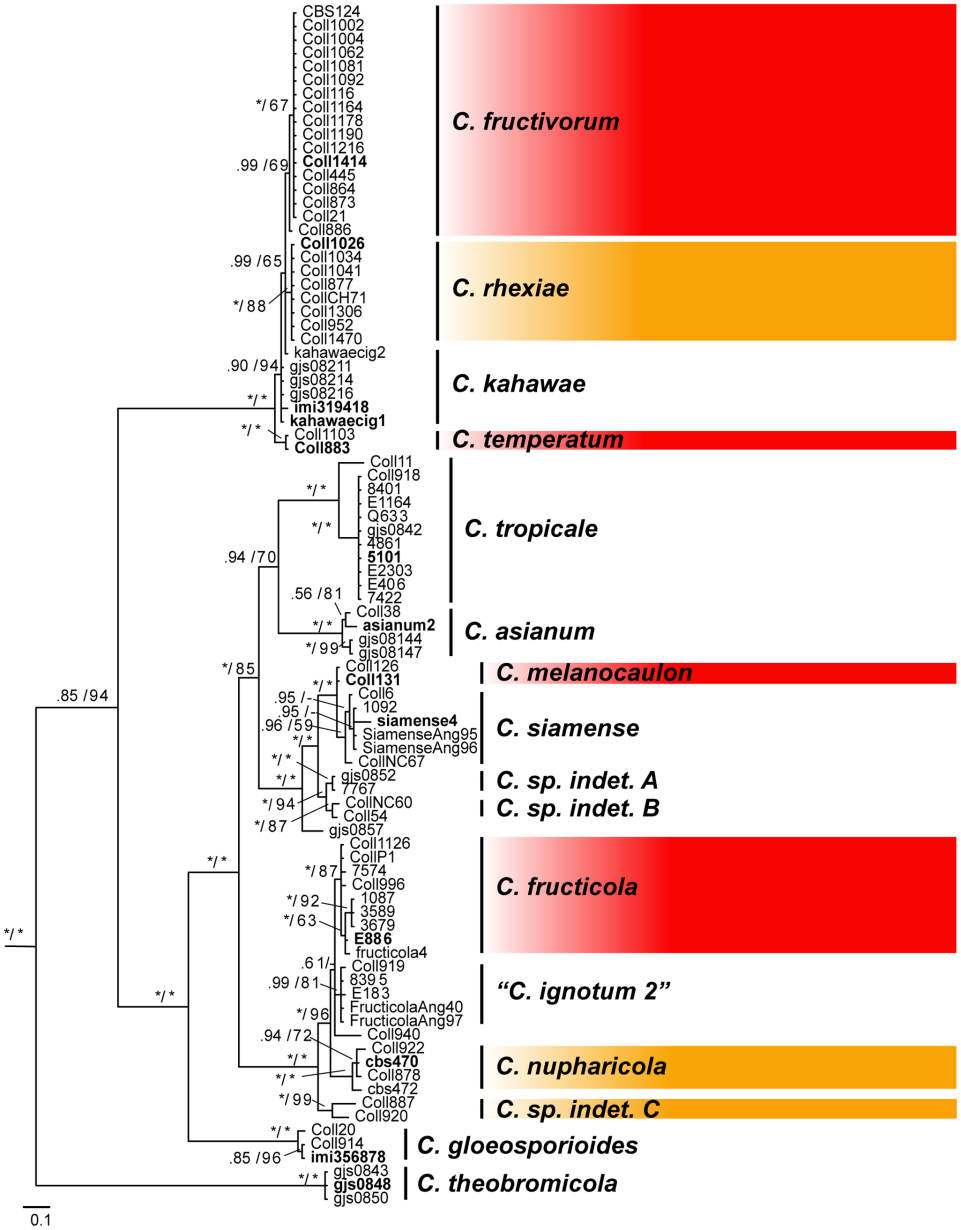
Bayesian majority rule consensus tree with support values (PP/ML-BS) for the combined analysis with expanded taxon sampling but a reduced dataset (D3G - nrITS, *btub*, and *apn2mat/IGS*). Clades enclosed in red boxes contain isolates from *Vaccinium macrocarpon* in cultivated cranberry beds in North America. Clades enclosed in orange boxes contain isolates from host species sympatric with cultivated *V. macrocarpon*.

The monophyly of species delimited on the basis of the combined analysis presented in Figure 1 is largely supported by results from the analysis of D3G, which includes three additional species and one subspecies (Figure 3). Alternate sister group relationships are suggested by this analysis, however branch support values are generally lower than those in Figure 1 and the backbone of the tree topology remains unchanged. The addition of sequence data from ex-type strains of *C. asianum* and *C. siamense* allowed for the identification of isolates in clades that include these species. The assignment of isolates Coll38, G.J.S. 08-144, and G.J.S. 08-147 to *C. asianum* and isolates Coll6, 1092, and NC67 to *C. siamense* in Figure 1 and Figure 2 are based on the results presented in Figure 3. The inclusion of sequences from the ex-type of *C. fructicola* confirms that *C. ignotum* is a synonym as reported by Weir et al. [16]. In addition, the inclusion of two sequences of *C. kahawae subsp. cigarro*, including the ex-type, illustrates its close affinity to *C. rhexiae* and *C. fructivorum*. Weir et al. [16] also identified an isolate from cranberry (CBS124) as *C. kahawae subsp. cigarro*, however our analyses indicate this isolate belongs to a phylogenetically distinct lineage.

### Genealogical Sorting Indices

The genealogical sorting index represents a quantitative assessment of the exclusive ancestry of each species in a set of bootstrap trees for each individual locus and in the combined dataset. Terminal labels for this analysis match the species assignments based on the combined four-marker analysis (D4G), as previously described. The results of these analyses are presented in Table 3 and the taxon assignments are presented in Table 4.

**Table 3:** Genealogical sorting indices of 1000 bootstrap trees calculated using genealogical Sorting R package with 1,000 permutations.

Taxon	combined		nrITS		*tub2*		*apn2*		*apn2/mat12IGS*	
	*gsi_T_*	p	*gsi_T_*	p	*gsi_T_*	p	*gsi_T_*	p	*gsi_T_*	p
*kahawae*	0.740	***	0.155	***	0.073	NS	0.110	*	0.572	***
*fructivorum*	1.000	***	0.370	***	0.466	***	0.703	***	1.000	***
*rhexiae*	1.000	***	0.381	***	0.179	**	1.000	***	1.000	***
*temperatum*	1.000	**	0.404	**	1.000	***	0.494	**	0.901	***
*tropicale*	1.000	***	0.149	***	1.000	***	0.810	***	1.000	***
*CspindetA*	1.000	***	1.000	***	1.000	**	1.000	***	1.000	***
*melanocaulon*	1.000	**	0.022	NS	0.187	*	1.000	***	0.241	*
*CspindetB*	0.952	***	0.658	***	0.220	**	0.658	***	0.487	**
*CspindetC*	1.000	***	0.022	NS	0.114	*	0.986	***	1.000	***
*CspindetD*	1.000	**	0.022	NS	0.679	***	1.000	**	0.812	***
*ignotum*	1.000	***	0.221	***	0.751	***	0.757	***	1.000	***
*nupharicola*	1.000	***	0.198	***	1.000	***	1.000	***	1.000	***
*ignotum2*	1.000	***	0.064	*	1.000	***	1.000	***	0.447	***
*CspindetE*	1.000	**	0.024	NS	1.000	***	1.000	***	0.786	**
*gloeosporioides*	1.000	***	1.000	***	1.000	***	1.000	***	1.000	***
*theobromicola*	1.000	***	1.000	***	1.000	***	1.000	***	1.000	***

*** p-value <0.001.

** p-value <0.01.

* p-value <0.05.

NSp-value >0.05.

**Table 4:** Taxon assignments for the genealogical sorting index analysis.

Strain	Assignment	Strain	Assignment
CBS124	*fructivorum*	3679	*fructicola*
Coll1002	*fructivorum*	7574	*fructicola*
Coll1004	*fructivorum*	E886	*fructicola*
Coll1062	*fructivorum*	Coll919	“ignotum 2″
Coll1081	*fructivorum*	8395	“ignotum 2″
Coll1092	*fructivorum*	E183	“ignotum 2″
Coll116	*fructivorum*	GJS08211	*kahawae*
Coll1164	*fructivorum*	GJS08214	*kahawae*
Coll1178	*fructivorum*	GJS08216	*kahawae*
Coll1190	*fructivorum*	IMI319418	*kahawae*
Coll1216	*fructivorum*	Coll38	*asianum*
Coll1414	*fructivorum*	GJS08144	*asianum*
Coll21	*fructivorum*	GJS08147	*asianum*
Coll445	*fructivorum*	Coll878	*nupharicola*
Coll864	*fructivorum*	Coll922	*nupharicola*
Coll873	*fructivorum*	CBS470	*nupharicola*
Coll886	*fructivorum*	CBS472	*nupharicola*
Coll1103	*temperatum*	Coll1026	*rhexiae*
Coll883	*temperatum*	Coll1034	*rhexiae*
Coll126	*melanocaulon*	Coll1041	*rhexiae*
Coll131	*melanocaulon*	Coll1306	*rhexiae*
Coll887	*C. sp. indet. C*	Coll877	*rhexiae*
Coll920	*C. sp. indet. C*	Coll952	*rhexiae*
Coll940	Coll940	Coll1038	*rhexiae*
Coll6	*siamense*	Coll1470	*rhexiae*
CollNC67	*siamense*	GJS0843	*theobromicola*
1092	*siamense*	GJS0848	*theobromicola*
Coll54	*C. sp. indet. B*	GJS0850	*theobromicola*
CollNC60	*C. sp. indet. B*	Coll11	*tropicale*
GJS0852	*C. sp. indet. A*	Coll918	*tropicale*
7767	*C. sp. indet. A*	GJS0842	*tropicale*
GJS0857	GJS0857	4861	*tropicale*
Coll20	*gloeosporioides*	5101	*tropicale*
Coll914	*gloeosporioides*	7423	*tropicale*
IMI356878	*gloeosporioides*	8401	*tropicale*
Coll1126	*fructicola*	E1164	*tropicale*
Coll996	*fructicola*	E2303	*tropicale*
CollP1	*fructicola*	E406	*tropicale*
1087	*fructicola*	Q633	*tropicale*
3589	*fructicola*		

*Colletotrichum gloeosporioides, C. asianum,* and *C. theobromicola* reached the maximum ensemble *gsi* (*gsi_T_*) value of 1 when calculated across all bootstrapped trees from the nrITS data. Several species including *C. kahawae*, *C. tropicale, C. fructicola, C. nupharicola,* and “*C. ignotum 2″* had low, but significant, *gsi_T_* values based on the nrITS trees ranging from 0.064 to 0.221. Four species, *C. fructivorum, C. rhexiae, C. temperatum,* and *C. siamense* had moderate values of *gsi_T_* ranging from 0.370 to 0.658. The remaining species, *C. melanocaulon, C. sp. indet. C, C. sp. indet. B,* and *C. sp. indet. A* had non-significant *gsi_T_* values across the nrITS bootstrapped trees.

Unlike the *gsi_T_* values of the nrITS trees, all values calculated from the *tub2* trees were significant with the exception of *C. kahawae. Colletotrichum temperatum, C. tropicale, C. asianum, C. nupharicola,* “*C. ignotum 2″, C. sp. indet. C, C. gloeosporioides,* and *C. theobromicola* reached the maximum *gsi_T_* value of 1 when calculated across all bootstrapped trees. *Colletotrichum rhexiae, C. melanocaulon, C. siamense,* and *C. sp. indet. B,* had low but significant *gsi_T_* values ranging from 0.114 to 0.220. *Colletotrichum fructivorum, C. sp. indet. A* and *C. fructicola* had moderate to high *gsi_T_* values ranging from 0.466 to 0.751.

All *gsi_T_* values of the *apn2* trees were significant. *Colletotrichum kahawae* had the lowest value of 0.11, while all others were moderate to high. *Colletotrichum rhexiae, C. asianum, C. melanocaulon, C. sp. indet. A, C. nupharicola,* “*C. ignotum 2″, C. sp. indet. C, C. gloeosporioides,* and *C. theobromicola* had the maximum *gsi_T_* value of 1 across all bootstrapped trees. *Colletotrichum fructivorum, C. temperatum, C. tropicale, C. siamense, C. sp. indet. B,* and *C. fructicola* had moderate to high *gsi_T_* values ranging from 0.494 to 0.986.

Similarly, all *gsi_T_* values of the *apn2mat/IGS* trees were significant. *Colletotrichum melanocaulon* had the lowest value of 0.241, while all others were moderate to high. *Colletotrichum fructivorum, C. rhexiae, C. tropicale, C. asianum, C. sp. indet. B, C. fructicola, C. nupharicola, C. gloeosporioides,* and *C. theobromicola* had *gsi_T_* values of 1 when calculated across all bootstrapped trees, while *C. kahawae, C. temperatum, C. siamense, C. sp. indet. A,* “*C. ignotum 2″,* and *C. sp. indet. C* had *gsi_T_* values ranging from 0.447 to 0.901.

All *gsi_T_* values of trees from the combined dataset were significant with high values ranging from 0.740 to 1. The only species that did not reach the maximum possible *gsi_T_* value of 1 were *C. kahawae* (0.740) and *C. siamense* (0.952). All values based on the combined four-marker dataset represent strong measures of genealogical divergence across all 1000 bootstrap replicates.

### Taxonomy and Morphology

**Figure 4:**
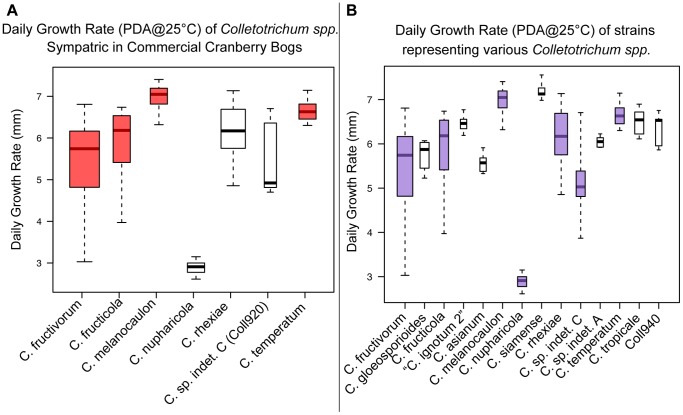
Box plots of daily growth rates representing various species in the *Colletotrichum gloeosporioides* species complex. A) Box plot of species isolated from *Vaccinium macrocarpon* and other sympatric host species in commercial cranberry beds. Red boxes represent species where strains have been isolated from *V. macrocarpon* in commercial beds only. Non-colored boxes represent species isolated only from sympatric host species. B) Box plot of several strains representing species within the *C. gloeosporioides* species complex. Purple boxes represent species that contain strains isolated from host plants found in wild and commercial cranberry bogs in North America. Non-colored boxes represent species not found in these habitats.

**Figure 5:**
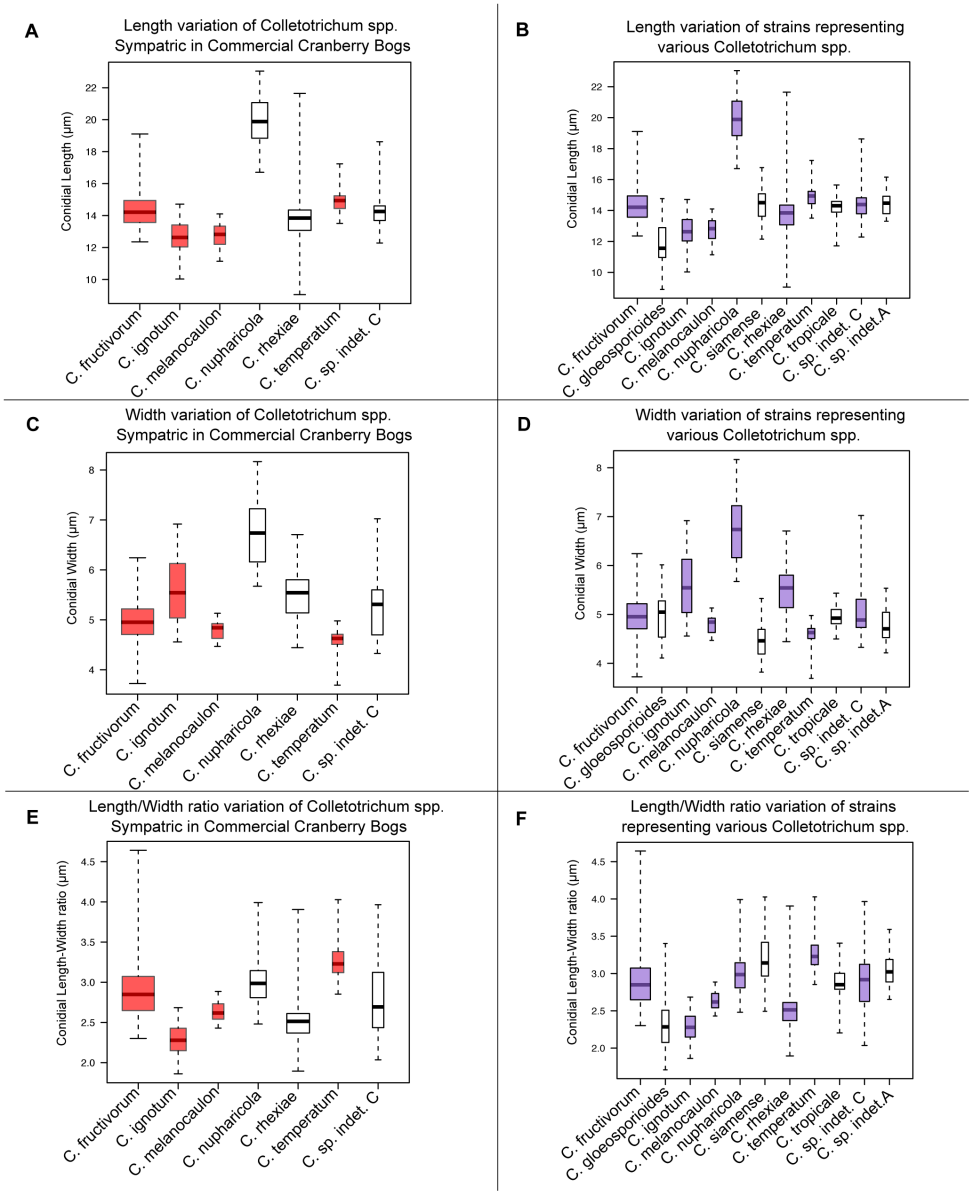
Box plots of conidial sizes representing various species in the *Colletotrichum gloeosporioides* species complex. A, C, E) Box plot of species isolated from *Vaccinium macrocarpon* and other sympatric host species in commercial cranberry beds. Red boxes represent species where strains have been isolated from *V. macrocarpon* in commercial beds only. The measurements for *C. sp. indet. C* were taken from the only strain to have been isolated from commercial cranberry beds. Non-colored boxes represent species isolated only from sympatric host species. B, D, F) Box plot of several strains representing species within the *C. gloeosporioides* species complex. Purple boxes represent species that contain strains isolated from host plants found in wild and commercial cranberry bogs in North America. The measurements for *Colletotrichum sp. indet. C* were taken from both known strains. Non-colored boxes represent species not found in these habitats.

**Figure 6:**
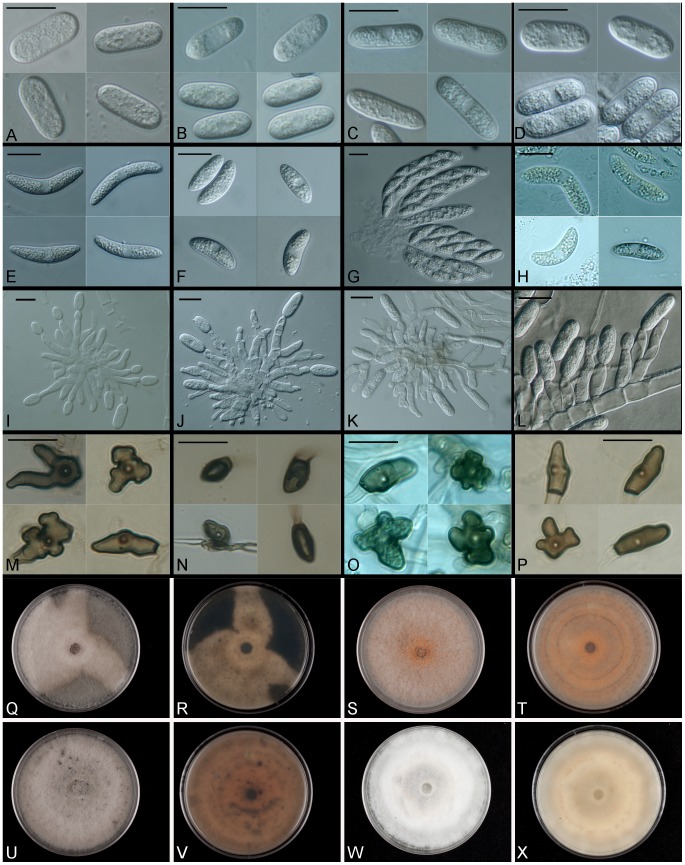
New and epitypified species of *Colletotrichum* from North American cranberry bogs. A-D. Conidia. A) *C. rhexiae* on CMA. B) *C. melanocaulon* on CMA. C) *C. temperatum* on CMA. D) *C. fructivorum* on CMA. E-H. Asci and ascospores. E) *C. rhexiae* on PDA. F) *C. temperatum* on CMA. G) *C. temperatum* on CMA. H) *C. fructivorum* on V8 agar. I-L. Phialides. I) *C. rhexiae* on CMA. J) *C. melanocaulon* on SNA. K) *C. temperatum* on CMA. L) *C. fructivorum* on CMA. M-P. Hyphal appressoria. M) *C. rhexiae.* N) *C. melanocaulon.* O) *C. temperatum*. P) *C. fructivorum*. Q-X. Colonies on PDA after 10 days at 25°C in the dark (100×15 mm plates). Q) *C. rhexiae* obverse. R) *C. rhexiae* reverse. S) *C. melanocaulon* obverse. T) *C. melanocaulon* reverse. U) *C. temperatum* obverse. V) *C. temperatum* reverse. W) *C. fructivorum* obverse. X) *C. fructivorum* reverse. All scale bars = 10 µm.

**Figure 7:**
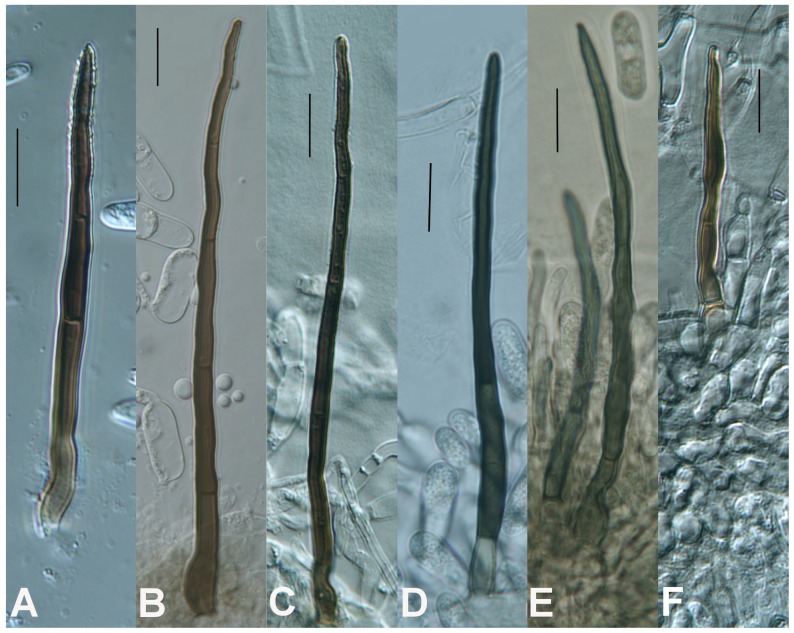
Setae. A) Colletotrichum melanocaulon on SNA. B) Colletotrichum rhexiae on CMA. C) Colletotrichum rhexiae on SNA. D) Colletotrichum fructivorum on CMA. E) Colletotrichum temperatum on CMA. F) Colletotrichum sp. indet. B on SNA. Scale bars = 10 µm.

Multilocus phylogenetic analysis of *Colletotrichum gloeosporioides s.l.* strains isolated from *V. macrocarpon* and other sympatric host species revealed several distinct lineages within the species complex. Comparison of growth rates and conidial morphology indicates that there is significant morphological overlap between *Colletotrichum* species isolated from *V. macrocarpon* and sympatric host species as well as between strains representing additional species within the species complex. *Colletotrichum nupharicola* is exceptional, exhibiting a very slow growth rate, with significantly longer and wider conidia than all other species included in this study. Comparisons of growth rates and conidial dimensions are presented in Figure 4 and Figure 5 respectively. Three new species are described below and *C. rhexiae* is epitypified. Conidial, ascospore, and colony morphology are represented in Figure 6 for each new species and *C. rhexiae*. Seta morphology is depicted in Figure 7.

*Colletotrichum fructivorum* V. Doyle, P.V. Oudem. & S.A. Rehner, sp. nov.

Index Fungorum LSID: urn:lsid:indexfungorum.org:names:801462.

MycoBank MB 801462 (Figure 6, Figure 7).

Similar to *Colletotrichum rhexiae* Ellis & Everh. but setae less abundant, the interquartile range of the ascospore length to width ratio smaller (3.2–3.9 um) and the interquartile range of the conidial length to width ratio larger on CMA. Common fruit-rot pathogen of cranberry (*Vaccinium macrocarpon* Aiton) in commercial production.

Growth rate (3.0–) 5.4 (–6.8) mm per day with standard deviation of 1.1 mm on PDA at 25°C [n = 54]; aerial mycelium floccose, white to greyish white, medium grey and brownish grey in some strains; sectoring common. Perithecia developing and maturing on V8 agar, clustered or solitary, dark brown to black, globose to obpyriform to papillate; ascospores allantoid, olive yellow, (15.7–) 16.6–18.2–18.5 (–26.9)×(3.8–) 4.5–5.1–5.6 (–7.1) µm with length/width ratio (2.6–) 3.2–3.7–3.9 (–5.6) µm [n = 30]. On CMA and SNA perithecial fundaments with melanized hyphae radiating from the base but mature ascospores not observed. On SNA aerial mycelium flocculose; conidial masses flesh to light orange, (12.4–) 13.2–14.3–15.2 (–18.6)×(3.8) 4.3–4.9–5.6 (–6.2) µm with length/width ratio (2.0–) 2.4–3.0–3.5 (–4.3). On CMA mycelium barely visible; conidial masses usually abundant, embedded in the medium and on the surface, flesh to light orange; phialides tapering towards the tip, monoblastic, (9.0–) 10.5–12.9–14.2 (–18.1)×(2.9–) 3.7–3.9–4.1 (–4.9) µm [n = 30] at the widest point; conidia subcylindrical to slightly tapered with obtuse apices, (12.4–) 13.6–14.3–14.9 (–19.1)×(3.7–) 4.7–5.0–5.2 (–6.2) µm with length/width ratio (2.3–) 2.7–2.9–3.1 (–4.6) µm [n = 240]; hyphal appressoria melanized, clavate or irregular, shallowly lobed, terminal, (3.9–) 8.0–9.4–10.8 (–15.2)×(3.1–) 3.9–4.5–4.7 (–7.7) µm with length/width ratio (0.6–) 1.8–2.2–2.6 (–3.5) µm [n = 30].

#### Habitat and Distribution

Commonly isolated as a fruit-rot pathogen and from asymptomatic tissue of *Vaccinium macrocarpon* in commercial cranberry beds throughout North America. Also isolated from *Rhexia virginica* from asymptomatic infections growing in commercial cranberry beds and symptomatic fruit of *V. oxycoccos* in a wild cranberry bog in Pennsylvania.

#### Etymology

The specific epithet, “fructivorum”, refers to the propensity of the species to be associated with fruit-rot of cranberry. From the Latin *fructus,* fruit, and *–vorous*, eating.

#### Holotype

USA. New Jersey: isolated as a fruit-rot pathogen of *Vaccinium macrocarpon,* Burlington County, 39.8128 N, 74.6399 W, Oct 2010, *V. Doyle, P.V. Oudemans, C. Constantelos Coll1414* (BPI 884103, CBS 133125).

#### Additional specimens examined

USA. New Jersey: isolated as a stem endophyte of *V. macrocarpon,* Burlington County, 39.9518 N, 74.5005 W, Oct 2010, *V. Doyle Coll1092* (BPI 884114, CBS 133135); isolated from *Rhexia virginica,* Burlington County, 39.7348 N, 74.5120 W, Sept 2009, *V. Doyle Coll864* (BPI 884108, CBS 133130); isolated as a fruit-rot pathogen of *V. macrocarpon*, Burlington County, 39.9518 N, 74.5005 W, Oct 2009, *V. Doyle, P.V. Oudemans Coll445* (BPI 884105, CBS 133127); isolated as a fruit-rot pathogen of *V. macrocarpon*, Burlington County, Fall 2007, *P.V. Oudemans* GLO7-17 (BPI 884111, culture *V. Doyle Coll21* = CBS 133133); Collected by C.L. Shear, deposited to CBS in Apr 1922 (CBS124.22). Massachusetts: isolated as a fruit-rot pathogen of *V. macrocarpon*, Plymouth County, Nov 2010, *V. Doyle Coll1062*; isolated as a fruit-rot pathogen of *V. macrocarpon*, Plymouth County, Nov 2010, *V. Doyle Coll1081* (BPI 884099, CBS 133121); isolated as a fruit-rot pathogen of *V. macrocarpon*, *P.V. Oudemans* (culture *V.Doyle Coll116*). Wisconsin: isolated as a fruit-rot pathogen of *V. macrocarpon*, Monroe County, 44.0685 N, 90.4015 W, Sept 2010, *P.V. Oudemans, C. Constantelos, E. Zeldin* WI-1 (BPI 884104, culture *V. Doyle Coll1164* = CBS 133126); isolated as a fruit-rot pathogen of *V. macrocarpon*, Wood County, 44.3854 N, 90.0151 W, Sept 2010, *P.V. Oudemans, C. Constantelos, E. Zeldin* WI-53 (culture *V. Doyle Coll1216*); isolated as a fruit-rot pathogen of *V. macrocarpon*, Juneau County, 44.2072 N, 90.0995 W, Sept 2010, *P.V. Oudemans, C. Constantelos, E. Zeldin* WI-27 (culture *V. Doyle Coll1190*); Delaware: isolated as a fruit-rot pathogen of *V. macrocarpon,* Kent County, 39.3130 N, 79.5555 W, Fall 2009, *P.V. Oudemans, C. Constantelos* GLO9-1 (culture *V. Doyle Coll873*). Pennsylvania: isolated as a fruit-rot pathogen of *V. oxycoccos*, Monroe County, Tannersville Cranberry Bog Preserve, 41.0376 N, 75.2648 N, *V. Doyle Coll886* (BPI 884102, CBS 133124). CANADA. British Columbia: isolated as a fruit-rot pathogen of *V. macrocarpon*, Oct 2010, *V. Doyle, P.V. Oudemans, B. Mouza Coll1002* (BPI 884106, CBS 133128)*;* isolated as a fruit-rot pathogen of *V. macrocarpon*, Oct 2010, *V. Doyle, P.V. Oudemans, B. Mouza Coll1004;*

#### Notes

*Glomerella rufomaculans var. vaccinii* was described by C.L. Shear in 1907 [70] from leaves of *Vaccinium macrocarpon* in New Jersey. The type material was a slide (slide *no. 1447A C.L.S*.) of a single ascospore isolate made from this collection deposited in the “pathological collection of the Department of Agriculture”, now housed in the collections at the Systematic Mycology and Microbiology Laboratory in Beltsville, Maryland (BPI). While other slides designated by C.L. Shear as type material for new species published in the same protologue were located at BPI, slide *no. 1447A C.L.S.* was not located and is thought to be lost. However, a culture deposited by Shear in April, 1922 to Centraalbureau voor Schimmelcultures in the Netherlands was obtained, sequenced and found to be conspecific with other isolates described here. The nomenclature used by CBS in designating this isolate as *Glomerella rufomaculans-vaccinii,* however, seems to be based on a typographical error as C.L. Shear clearly intended recognition of this taxon at the varietal level and there is no indication in the literature that it has been formally elevated to species. Furthermore, *Glomerella rufomaculans* was subsumed into *Glomerella cingulata* (anamorph: *Colletotrichum gloeosporioides*) with the revisionary work of von Arx in 1957 [11], necessitating the designation of the new specific epithet, *Colletotrichum fructivorum.* We have chosen a recently isolated strain as the ex-type strain due to the lack of sporulation observed in the culture deposited by Shear and the fact the holotype designated here is a single ascospore isolate for which we have morphological data for both the anamorph and the teleomorph (Figure 6).

*Colletotrichum melanocaulon* V. Doyle, P.V. Oudem. & S.A. Rehner sp. nov.

Index Fungorum LSID: urn:lsid:indexfungorum.org:names:801464.

MycoBank MB 801464 (Figure 6, Figure 7).

Similar to *Colletotrichum gloeosporioides* (Penz.) Sacc. but associated with stem canker of *Vaccinium macrocarpon* Aiton.

Growth rate (4.9–) 6.4 (–7.3) mm per day with standard deviation of 0.8 mm on PDA at 25°C [n = 18]; aerial mycelium floccose, white to light grey, sectoring observed; fertile perithecia not observed; conidiomata forming abundantly in concentric rings in type strain, conidial masses orange. On SNA aerial mycelium flocculose; conidiomata abundant, conidial masses light orange; phialides tapering towards the tip, monoblastic, (8.6–) 11.5–13.6–15.3 (–19.0)×(2.1–) 2.8–3.3–3.9 (–4.8) µm [n = 34] at the widest point; conidia subcylindrical to slightly tapered with obtuse apices, (11.3–) 12.6–13.1–13.4 (–14.8)×(4.4–) 5.1–5.5–5.9 (–6.3) µm with length/width ratio (2.1–) 2.3–2.4–2.5 (–3.0) µm [n = 60]. On CMA mycelium barely visible, conidial masses pale orange to melon; phialides tapering toward the tip, monoblastic, (11.7–) 13.5–14.1–14.4 (–17.0)×(2.8–) 3.2–3.4–3.7 (–3.9) µm [n = 10] at the widest point; conidia subcylindrical to slightly tapered with obtuse apices, (11.1–) 12.2–12.7–13.3 (–14.1)×(4.5–) 4.7–4.8–4.9 (–5.1) µm with length/width ratio (2.4–) 2.6–2.6–2.7 (–2.9) µm [n = 30]; hyphal appressoria melanized, subglobose to clavate, not lobed, terminal, (4.4–) 5.8–6.3–7.2 (–8.2)×(3.3–) 4.1–4.5–4.9 (–5.9) µm with length/width ratio (1.0–) 1.1–1.4–1.6 (–1.9) µm [n = 10].

#### Habitat and Distribution

Isolated from stem canker lesions of *Vaccinium macrocarpon* in commercial cranberry beds in New Jersey.

#### Etymology

The specific epithet, “melanocaulon”, refers to the brown or black stems from which the species was isolated. From the Greek, *melano*-, black or very dark, and *caulon*, stem, in agreement with the *neuter* generic name *Colletotrichum.*

#### Holotype

USA. New Jersey: isolated from cankered stems of *Vaccinium macrocarpon,* Burlington County, 39.7539 N, 74.5387 W, Aug 2008, *V. Doyle Coll131* (BPI 884113, CBS 133251).

#### Additional specimens examined

USA. New Jersey: isolated from cankered stems of *Vaccinium macrocarpon,* Burlington County, 39.7538 N, 74.5400 W, Aug 2008, *V. Doyle Coll126* (BPI 884101, CBS 133123).

*Colletotrichum temperatum* V. Doyle, P.V. Oudem. & S.A. Rehner, *sp. nov.*

Index Fungorum LSID: urn:lsid:indexfungorum.org:names:801463.

MycoBank MB 801463 (Figure 6, Figure 7).

Similar to *Colletotrichum rhexiae* Ellis & Everh. and *C. fructivorum* but ascospore length smaller, and ascospore length to width ratio smaller.

Growth rate (6.3–) 6.6 (–7.2) mm per day with standard deviation of 0.2 mm on PDA at 25°C [n = 18]; aerial mycelium floccose, white to greyish white, sectoring observed. Mature ascospores not observed on PDA. On CMA aerial mycelium flocculose to barely visible; perithecia solitary to clustered, dark brown to black, subglobose to obpyriform, ascospores hemisphaeroid to reniform, olive brown, (13.5–) 14.3–14.7–15.3 (–17.1)×(4.6–) 5.3–5.5–5.7 (–5.8) µm with length/width ratio (2.3–) 2.6–2.7–2.8 (–3.3) µm [n = 22]; conidial masses abundant on surface and embedded in agar, orange yellow; phialides tapering toward the tip, monoblastic, (7.7–) 11.3–13.9–15.7 (–22.6)×(2.2–) 3.3–3.4–3.7 (–4.2) µm [n = 30] at the widest point; conidia subcylindrical to slightly tapered, rarely medially constricted, with obtuse apices, (13.5–) 14.5–14.9–15.2 (–17.2)×(3.7–) 4.5–4.6–4.7 (–4.9) µm with length/width ratio (2.9–) 3.1–3.3–3.4 (–4.0) µm [n = 30]; hyphal appressoria melanized, irregular, shallowly to deeply lobed, terminal, (7.7–) 9.3–10.7–11.7 (–13.2)×(4.9–) 6.0–7.4–8.6 (–11.5) µm with length/width ratio (1.0–) 1.2–1.5–1.8 (–2.3) µm [n = 50].

#### Habitat and Distribution

Isolated from rotten fruit of an ornamental cultivar, *Vaccinium macrocarpon* ‘Hamilton’, growing at The New York Botanical Garden and as a stem endophyte of *V. macrocarpon* in a commercial cranberry bog in New Jersey.

#### Etymology

The specific epithet, “temperatum”, refers to the known distribution of the species. From the Latin, *temperatum*, temperate.

#### Holotype

USA. New York: isolated from rotten fruit of *Vaccinium macrocarpon,* Bronx County, The New York Botanical Garden, 40.8674 N, 73.8780 W, Nov 2009, *V. Doyle* & *C. Mozzicato Coll883* (BPI 884100, CBS 133122).

#### Additional specimens examined

USA. New Jersey: isolated as a stem endophyte of *Vaccinium macrocarpon,* Burlington County, 39.9514 N, 74.5008 W, Oct 2010, *V. Doyle Coll1103* (BPI 884098, CBS 133120).

*Colletotrichum rhexiae* Ellis & Everh. Proceedings of the Academy of Natural Sciences of Philadelphia 46∶372. 1894.

**Figure 8:**
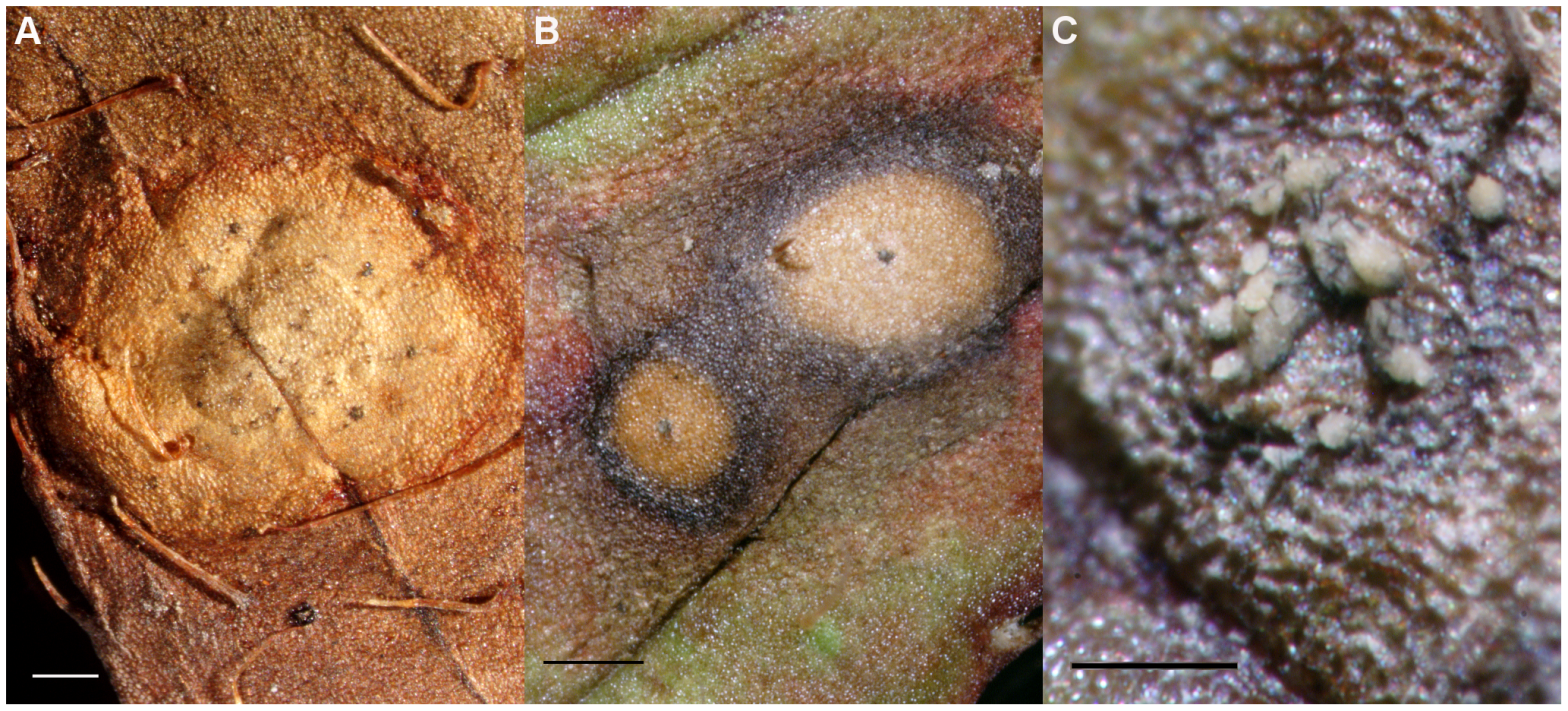
Acervuli of *Colletotrichum rhexiae* on *Rhexia virginica.* A) Acervulus of the holotype, *A. Commons 2534*, on leaf. Scale bar = 500 µm. B) Acervuli on leaves of *R. virginica.* Scale bar = 250 µm. C) Acervulus with setae and conidial masses from which *V. Doyle Coll1470* was isolated. Scale bar = 250 µm.

MycoBank MB 178511 (Figure 6, Figure 7, Figure 8).

Growth rate (4.9–) 6.2 (–7.1) mm per day with standard deviation of 0.6 mm on PDA at 25°C [n = 36]; aerial mycelium floccose, white to light grey to greyish brown, sectoring common; perithecia solitary to clustered, dark brown to black, ascospores fusiform to lunate, greyish yellow (18.1–) 21.7–23.2–24.4 (–27.5)×(3.5–) 3.9–4.1–4.3 (–4.7) µm with length/width ratio (4.5–) 5.2–5.8–6.4 (–7.6) µm [n = 60]; On SNA aerial mycelium flocculose; conidial masses flesh to light orange, phialides tapering toward the tip, monoblastic, (9.5–) 13.5–14.7–16.2 (–17.6)×(3.0–) 3.6–3.7–4.0 (–4.4) µm [n = 15] at the widest point; conidia subcylindrical with obtuse apices, (11.7–) 13.4–14.0–14.3 (–27.9)×(3.9–) 5.4–5.8–6.4 (–7.4) µm with length/width ratio (1.8–) 2.1–2.5–2.6 (–4.4) µm [n = 60]. On CMA mycelium barely visible; conidial masses abundant on the surface, flesh to light orange, phialides tapering towards the tip, monoblastic, (5.9–) 10.9–13.4–15.0 (–28.9)×(2.6–) 3.3–3.7–4.2 (–4.7) µm [n = 30] at the widest point; conidia subcylindrical with obtuse apices, (9.1–) 13.1–13.7–14.4 (–21.7)×(4.4–) 5.1–5.5–5.8 (–6.7) µm with length/width ratio (1.9–) 2.4–2.5–2.6 (–3.9) µm [n = 120]; hyphal appressoria melanized, clavate, obclavate to fusiform and irregular, shallowly to deeply lobed, terminal, (7.0–) 9.4–10.9–12.8 (–15.2)×(3.9–) 5.0.–6.1–7.1 (–9.7) µm with length/width ratio (1.0–) 1.3–1.9–2.2 (–3.4) µm [n = 30].

#### Holotype

USA. Delaware: acervuli on several leaves of *Rhexia virginica*, Kimensi, Delaware, *A. Commons 2534.*

#### Epitype

isolated from lesioned stem tissue of *Rhexia virginica,* Sussex County, Delaware, Cape Henlopen State Park, 38.7778 N, 75.1062 W, Nov 2010, *V. Doyle Coll1026* (BPI 884112, CBS 133134).

**Additional Specimens Examined.** USA. Delaware: isolated from lesioned stem tissue of *Rhexia virginica,* Sussex County, Cape Henlopen State Park, 38.7772 N, 75.1075 W, Nov 2010, *V. Doyle Coll1038*; isolated from healthy fruit of *Vaccinium macrocarpon,* Sussex County, Cape Henlopen State Park, 38.7772 N, 75.1072 W, Nov 2009, *V. Doyle, P.V. Oudemans, C. Constantelos Coll877* (BPI 884110, CBS 133132); isolated from rotten fruit of *Vaccinium macrocarpon,* Sussex County, Cape Henlopen State Park, 38.7770 N, 75.1075 W, Nov 2010, *V. Doyle Coll1034* (BPI 884115, CBS 133136); isolated from rotten fruit of *Vaccinium macrocarpon,* Sussex County, Cape Henlopen State Park, 38.7772 N, 75.1075 W, Nov 2010, *V. Doyle Coll1041*. New Jersey: isolated from stem tissue of *R. virginica* in commercial cranberry bog, Burlington County, 39.7546 N, 74.5388 W, Sept 2010, *V. Doyle Coll952* (BPI 884109, CBS 133131)*;* isolated from stem tissue of *R. virginica* in commercial cranberry bog, Burlington County, 39.7309 N, 74.5160 W, July 2010, *V. Doyle Coll1306*. Maryland: isolated from leaf anthracnose lesions of *R. virginica,* Prince George’s County, 39.0308 N, 76.7886 W, Sept 2011, *V. Doyle* & *S.A. Rehner Coll1470* (BPI 884107, CBS 133129).

#### Notes

*Colletotrichum rhexiae* was described from leaves of *Rhexia virginica* collected in “Kimensi”, Delaware in 1894. We attempted to find *R. virginica* in an area thought to be the type locality but were unable to find any host plants. The type material, consisting of *R. virginica* leaves with circular lesions, is morphologically consistent *in vitro* with material collected in Cape Henlopen State Park in Delaware on *R. virginica* and is conspecific with material isolated from circular lesions on the same host in Maryland (Figure 8). We have decided to epitypify *C. rhexiae* with a living culture based on the necessity of molecular data for species delimitation in *Colletotrichum* and the need to evaluate morphological characters from a living culture.

## Discussion

### Sympatric Lineages and Host Distribution in North American Cranberry Bogs

Several phylogenetic studies have confirmed the ability of multiple lineages within *Colletotrichum gloeosporioides s.l.* to colonize the same host family, genus, or species (e.g. *Coffea arabica* L. [58]; *Jasminum sambac* (L.) Aiton [71]; *Hemerocallis spp.* [72]; Amaryllidaceae [73]). Seven well-supported lineages of *C. gloeosporioides s.l.* were isolated from North American cranberry bogs, either from *V. macrocarpon, V. oxycoccos,* or other sympatric host plant species (Table 1 and Figure 1). Similar to the results from previous studies focused on a single host or group of closely related hosts, multiple lineages within the species complex can be isolated from *Vaccinium spp.*, including five species (*C. fructivorum, C. rhexiae, C. temperatum, C. melanocaulon,* and *C. fructicola*) as endophytes and/or pathogens of *V. macrocarpon*, two lineages (*C. fructivorum* and *C. sp. indet. C*) from *V. oxycoccos,* and one species (*C. fructicola*) from *V. corymbosum*. Likewise, three species have been found to colonize *Rhexia virginica* L., an herbaceous perennial inhabiting well-drained soils that are seasonally inundated; often found sympatric with *V. macrocarpon* in wild and agricultural habitats in eastern North America.

In contrast, a single lineage has been isolated from *Nuphar* and *Nymphaea* within and beyond North American cranberry bogs. *Colletotrichum nupharicola* was previously described from *Nuphar lutea subsp. polysepala* (Engelm.) E.O. Beal in Washington and Idaho and *Nymphaea odorata* Aiton in Rhode Island. Our study indicates that this species is also present in irrigation reservoirs in agricultural cranberry beds in New Jersey, growing on *Nuphar lutea* (L.) Sm. The morphological distinctiveness of *C. nupharicola* makes it one of the few species within the species complex that can be reliably identified on the basis of conidial and cultural morphology and thus can be more easily tracked. *Colletotrichum nupharicola* was frequently encountered on *Nuphar* during the course of this study; however, other lineages were not isolated from either *Nuphar* or *Nymphaea*.

*Chamaecyparis thyoides* (L.) Britton, Sterns & Poggenb. is a common and dominant tree species adjacent to cranberry bogs in parts of eastern North America and can be found as a weed in commercial cranberry beds. Bills and Polishook [25] reported *C. gloeosporioides s.l.* from *Chamaecyparis thyoides* collected near cranberry bogs in eastern North America, however they reported just two isolates in a survey of endophytes of the host in New Jersey. A single isolate of *C. gloeosporioides s.l.* was isolated from *C. thyoides* in this study and found to be sister to *Coll887*, isolated from a diseased fruit of *V. oxycoccos* in West Virginia. These isolates form a phylogenetically distinct but undescribed lineage (*C. sp. indet. C*). Given the apparent ecological distinctiveness, geographical separation, and relative sequence dissimilarity of these isolates, this lineage will remain undescribed until additional, closely related isolates are collected.

### Host Preference, Host Organ Preference, and Habitat Distribution

Species of *C. gloeosporioides s.l.* are largely considered to be host generalists with few exceptions (such as *C. salsolae* [16]). There are other species that have been isolated from single host species or genera, but these have been sampled primarily from cultivated plants where isolates may be transferred with plant material or from a narrow geographical range in native habitats (e.g. *C. ti* [16]; *C. psidii* [16]). Similarly, strains isolated from *V. macrocarpon* are distributed throughout the *C. gloeosporioides* species complex, with little indication of host specificity for most lineages. Two of those lineages, *C. temperatum* and *C. melanocaulon*, originate solely from *V. macrocarpon,* however these were isolated only from cultivated habitats and given further sampling may be found on additional hosts. *Colletotrichum temperatum* has been isolated as a stem endophyte of *V. macrocarpon* and from rotten fruit in a horticultural variety (*V. macrocarpon* cv. Hamilton: not grown for agricultural production). The other species, *C. melanocaulon*, is associated with an emerging stem canker disease on cranberry where it has been isolated from affected stem tissue collected from field populations in commercial cranberry beds. This study shows, however, that a composite of host, habitat, and host organ origin can be a useful indicator of lineage identity in some cases.

Isolates of *C. fructivorum* and *C. rhexiae* are not host specific; *C. fructivorum* has been isolated from *V. macrocarpon, V. oxycoccos* and *R. virginica*, while *C. rhexiae* has been isolated from *V. macrocarpon* and *R. virginica*. Nevertheless, the fact that all isolates originating from *V. macrocarpon* in wild habitats are conspecific with *C. rhexiae,* and all isolates from diseased fruit in cranberry agricultural production areas are conspecific with *C. fructivorum,* suggests that host, host organ, and habitat can be useful indicators of lineage identity. Gonzalez et al. [33] noted a correlation between organ-specific pathogenicity and genotype in isolates from the *C. gloeosporioides* complex. Given their findings, it appears selection for pathogenicity to fruit may be enabling *C. fructivorum* to colonize fruit to the exclusion of all other lineages within the species complex that can be found in commercial cranberry beds.

Episodic selection has been proposed to be a common force driving divergence among fungal species [74]–[76]. Host shifts are prominent among the factors responsible for episodic selection and speciation, and has recently been proposed for *C. kahawae* [59]. In contrast, habitat transformation has been implicated as a factor contributing to population divergence in *Colletotrichum cereal*e, a broadly distributed turfgrass pathogen [77]. The broad distribution of *C. fructivorum* in commercial cranberry beds and restriction to cultivated habitats suggests that the shift to agricultural production of cranberry may have led to its divergence from *C. rhexiae.* However, this conclusion is preliminary and would better be addressed with additional sampling and more variable molecular markers.

The only North American lineage of *C. gloeosporioides s.l.* sympatric with cranberry with some indication of host preference over a broad geographical range is *C. nupharicola.* While *C. nupharicola* has been isolated from two aquatic host genera, *Nuphar* and *Nymphaea,* both of these genera are in the same family, Nymphaeaceae, indicating host specificity at the family level. This species is broadly distributed on Nymphaeaceae from Alaska through the Rocky Mountains to eastern North America (Dennis A. Johnson, pers. communication, and V. Doyle, unpublished data).

### Geographic and Host Distribution of *Colletotrichum fructivorum*

This and other studies of *Colletotrichum gloeosporioides s.l.* have demonstrated the utility of delimiting species using multi-locus DNA sequence data (e.g. [14], [16]). However, given the cryptic nature of many species within the complex and the apparently broad diversity of species at small spatial scales it is difficult to design an appropriate sampling scheme to capture the species diversity of *Colletotrichum* within a given host, habitat or geographic range. To develop a better understanding of scenarios that promote species divergence, it is necessary to understand the factors that lead to population substructure within a focal species. One of the objectives of this study was to identify one or more broadly distributed lineages within the *C. gloeosporioides* species complex that occur on cranberry to serve as a system for investigation of the factors driving the divergence between populations. Given the widespread occurrence of *Colletotrichum gloeosporioides s.l.* as the causal agent of fruit-rot in cranberry agricultural areas, the economic importance of cranberry production in North America, and the ease at which *C. gloeosporioides s.l.* can be isolated from diseased fruit in commercial cranberry production areas, we sampled diseased fruit across several of the major cranberry production areas in North America and Canada (Table 1). All strains of *C. gloeosporioides s.l.* isolated from fruit in commercial cranberry production areas were found to belong to *C. fructivorum* regardless of geographic origin. This reveals that *C. fructivorum* is broadly distributed in North America from Delaware to Massachusetts and west to Washington and British Columbia. In addition, there is evidence that strains of *C. fructivorum* are not organ specific (fruit and stems) and not host specific (*V. macrocarpon, V. oxycoccos,* and *R. virginica*). The combination of economic importance, broad geographic distribution, and diverse organ and host association makes this a suitable species for examining biotic and abiotic factors that influence microevolutionary processes in *C. gloeosporioides s.l.*

### Diversity of North American Lineages

Fungal strains included in this study have been placed in a broader phylogenetic context to better understand the relationships among species within the *Colletotrichum gloeosporioides* species complex. Despite the fact that there is a significant body of research on North American isolates thought to be members of the species complex (e.g. [8], [23], [24], [27], [33], [78]–[83]) there is a paucity of modern systematic research addressing the phylogenetic and geographic distribution of North American lineages of *C. gloeosporioides s.l.* However, a recent study by Weir et al. [16] included several North American isolates.

With the inclusion of *C. rhexiae, C. temperatum, C. melanocaulon,* and *C. asianum,* our research indicates there is a broader diversity of species in North America than has previously been reported. The species that have been isolated from native North American plant species include *C. aeschonymenes*, *C. asianum, C. clidemiae, C. fructicola, C. fructivorum, C. melanocaulon, C. nupharicola, C. rhexiae, C. temperatum, C. theobromicola,* and *C. “f. sp. camelliae”*, while *C. gloeosporioides, C. musae,* and *C. siamense* have been reported from introduced host species. However, there has been very little work addressing the geographic distribution of the species complex in North America. The only species that have been isolated from more than a very localized range are *C. fructivorum*, *C. rhexiae,* and *C. nupharicola*. Our understanding of the importance of the *C. gloeosporioides* species complex in North America will continue to improve as more North American mycologists and plant pathologists begin to place their isolates in the appropriate phylogenetic context.

*Colletotrichum rhexiae* and *C. fructivorum* each present a unique opportunity to investigate the role of host and geography in shaping the microevolutionary patterns that may ultimately lead to species divergence. *Colletotrichum rhexiae* was described by Ellis and Everhart in the late 1800’s [18] from Delaware while *C. fructivorum* has been known from the same region since at least 1907 with its formal recognition by Shear [19], [70] as *Glomerella rufomaculans var. vaccinii* and likely earlier as implicated by the work of Byron Halsted [18]. These species have apparently been sympatric for at least 105 years. While we do not have DNA evidence that *C. rhexiae* has been present for this extent of time, we have examined the original type material and it is not apparently distinct from our collections of *C. rhexiae* in New Jersey, Delaware and Maryland. In contrast, we do have a culture deposited to CBS in 1922 by Shear of what he considered to be *G. rufomaculans var. vaccinii* and it is identical in sequence across 4 loci to isolates collected from cranberry fruit in 5 US states and BC, from cranberry stem in New Jersey, and from *Rhexia virginica* growing as a weed in agricultural cranberry beds. The presence of sexual reproductive structures in both species suggests the potential for genetic exchange among conspecific individuals (unless they are obligately homothallic). However, despite their sympatry and ability to reproduce sexually, strong statistical support at the bifurcation between *C. rhexiae* and *C. fructivorum* indicates the presence of other biological factors reinforcing species limits. However, an objective test of species boundaries is better approached with more rapidly evolving markers variable within species, such as microsatellites, and using more appropriate non-phylogenetic algorithms to determine if there is any evidence of introgression. Further investigation using more rapidly evolving genetic markers could also lend the potential for inferring the reproductive strategy of these species and better understand the factors influencing species divergence within *Colletotrichum*. In addition, with the advancement of next-generation sequencing technology, it is possible to investigate these sister species at the genomic level to understand how genomic modifications (gene divergence, content/turnover or synteny) may influence pathogenicity on different hosts or in distinct organs.

## Conclusions

*Colletotrichum gloeosporioides s.l.* has long been implicated as a pathogen of cranberry in commercial production areas, but a more refined understanding of the species responsible for fruit-rot of cranberry has not been possible due to the difficulty of species delimitation using morphological features. This study resolves this issue, identifying *C. fructivorum* as the species associated with fruit-rot in cranberry. This study also lays the groundwork for future studies regarding the natural history and ecology of members of *C. gloeosporioides s.l.* and should provide useful tools for plant breeders and plant pathologists, in their efforts to develop resistant cultivars for an industry that utilizes one of North America’s few native crop species, *V. macrocarpon.* We have also determined that *C. fructivorum* is broadly distributed across North America and Canada in areas of commercial cranberry production and is capable of infecting alternate host species as well. This makes this species an appropriate model for addressing questions of population structure and dispersal at broad geographical and landscape level spatial scales. By virtue of a horizontal (among sympatric species) and vertical (among organs within a species) sampling scheme, we were able to uncover greater diversity of *C. gloeosporioides s.l.* in wild and commercial cranberry beds than has previously been suggested, revealing seven distinct lineages associated with *V. macrocarpon* and sympatric host species. Likewise, this level of sampling allowed us to determine that host specificity is not strongly implicated in determining macroevolutionary patterns among these species in temperate regions, with the exception of *C. nupharicola*. Similarly, despite the generalist nature of the species recovered in this study with respect to organ specificity, *C. fructivorum* does have an affinity for fruit of *V. macrocarpon* in commercial cranberry beds, to the exclusion of all other related species.

## Supporting Information

Figure S1 **Nelsen consensus from maximum parsimony analysis of nrITS gene from D4G.** Bootstrap support values shown above branches.
(TIF)

Figure S2 **Nelsen consensus from maximum parsimony analysis of partial beta-tubulin gene from D4G.** Bootstrap support values shown above branches.
(TIF)

Figure S3 **Nelsen consensus from maximum parsimony analysis of apn2 gene from D4G** ***.*** Bootstrap support values shown above branches.
(TIF)

Figure S4 **Nelsen consensus from maximum parsimony analysis of apn2/matIGS gene from D4G** ***.*** Bootstrap support values shown above branches.
(TIF)

Figure S5 **Maximum-likelihood majority-rule consensus tree of nrITS gene from D4G** ***.*** Bootstrap support values shown above branches. Outgroups (*C. aff. acutatum* and strains 4766, 3386, and 4801) have been trimmed from the tree. Branch lengths represent the mean across samples.
(TIF)

Figure S6 **Maximum-likelihood majority-rule consensus tree of partial beta-tubulin gene from D4G** ***.*** Bootstrap support values shown above branches. Outgroups (*C. aff. acutatum* and strains 4766, 3386, and 4801) have been trimmed from the tree. Branch lengths represent the mean across samples.
(TIF)

Figure S7 **Maximum-likelihood majority-rule consensus tree of apn2 gene from D4G** ***.*** Bootstrap support values shown above branches. Outgroups (*C. aff. acutatum* and strains 4766, 3386, and 4801) have been trimmed from the tree. Branch lengths represent the mean across samples.
(TIF)

Figure S8 **Maximum-likelihood majority-rule consensus tree of apn2/matIGS gene from D4G** ***.*** Bootstrap support values shown above branches. Outgroups (*C. aff. acutatum* and strains 4766, 3386, and 4801) have been trimmed from the tree. Branch lengths represent the mean across samples.
(TIF)

Figure S9 **Bayesian majority-rule consensus tree with support values (PP/ML-BS) resulting from the combined analysis of D3G+.**
(TIF)

Table S1 **GenBank accession numbers for sequence data generated in phylogenetic study of** ***C. gloeosporioides s.l.***
(DOCX)

Table S2 **GenBank accession numbers for sequence data generated in previous studies of** ***C. gloeosporioides s.l.***
(XLSX)

Text S1 **Discussion of the inferences drawn from the analysis of D3G+.**
(DOCX)

## References

[1] Morris CE, Bardin M, Kinkel LL, Moury B, Nicot PC, et al (2009) Expanding the Paradigms of Plant Pathogen Life History and Evolution of Parasitic Fitness beyond Agricultural Boundaries. PLoS Pathogens 5: 1–7. 10.1371/journal.ppat.1000693 PMC2790610 20041212

[2] Bailey JA, Jeger MJ, editors (1992) *Colletotrichum*: Biology, Pathology and Control: Wallingford, UK: CAB International.

[3] Latunde-Dada AO (2001) *Colletotrichum*: tales of forcible entry, stealth, transient confinement and breakout. Molecular Plant Pathology 2: 187–198. 20573006 10.1046/j.1464-6722.2001.00069.x

[4] Guozhong L, Cannon PF, Reid A, Simmons CM (2004) Diversity and molecular relationships of endophytic *Colletotrichum* isolates from the Iwokrama Forest Reserve, Guyana. Mycological Research 108: 53–63. 15035505 10.1017/s0953756203008906

[5] Hyde KD, Cai L, McKenzie EHC, Yang YL, Zhang JZ, et al (2009) *Colletotrichum*: a catalogue of confusion. Fungal Diversity 39: 1–17.

[6] Hyde KD, Cai L, Cannon PF, Crouch JA, Crous PW, et al (2009) *Colletotrichum* - names in current use. Fungal Diversity 39: 147–182.

[7] Sutton BC (1992) The genus *Glomerella* and its anamorph *Colletotrichum*. In: Bailey JA, Jeger MJ, editors. Colletotrichum: Biology, Pathology and Control. Surrey, UK: CAB International. 1–26.

[8] Oudemans PV, Caruso FL, Stretch AW (1998) Cranberry fruit rot in the Northeast: A complex disease. Plant Disease 82: 1176–1184. 10.1094/PDIS.1998.82.11.1176 30845403

[9] Adaskaveg JE, Forster H (2000) Occurrence and Management of Anthracnose Epidemics Caused by *Colletotrichum* Species on Tree Fruit Crops in California. In: Prusky D, Freeman S, Dickman MB, editors. *Colletotrichum*: Host Specificity, Pathology and Host-Pathogen Interaction. St. Paul, Minnesota: The American Phytopathogical Society Press. 317–336.

[10] Legard DE (2000) *Colletotrichum* diseases of Strawberry in Florida. In: Prusky D, Freeman S, Dickman MB, editors. Colletotrichum: Host Specificity, Pathology, and Host-Pathogen Interaction. St. Paul, Minnesota: American Phytopathological Society. 292–299.

[11] von Arx JA (1957) Die Arten der Gattung *Colletotrichum* Corda. Phytopathologische Zeitschrift 29: 414–468.

[12] Du M, Schardl CL, Nuckles EM, Vaillancourt LJ (2005) Using mating-type gene sequences for improved phylogenetic resolution of *Colletotrichum* species complexes. Mycologia 97: 641–658. 16392253 10.3852/mycologia.97.3.641

[13] Crouch JA, Clarke BB, Hillman BI (2006) Unraveling the evolutionary relationships among the divergent lineages of *Colletotrichum* causing anthracnose disease in turfgrass and corn. Phytopathology 96: 46–60. 18944204 10.1094/PHYTO-96-0046

[14] Rojas EI, Rehner SA, Samuels GJ, Van Bael SA, Herre EA, et al (2010) *Colletotrichum gloeosporioides s.l.* associated with *Theobroma cacao* and other plants in Panamá: multilocus phylogenies distinguish host-associated pathogens from asymptomatic endophytes. Mycologia 102: 1318–1338. 20943565 10.3852/09-244

[15] Silva DN, Talhinas P, Várzea V, Cai L, Paulo OS, et al (2012) Application of the *Apn2/MAT* locus to improve the systematics of the *Colletotrichum gloeosporioides* complex: an example from coffee (*Coffea* spp.). Mycologia 104: 396–409. 22086913 10.3852/11-145

[16] Weir BS, Johnston PR, Damm U (2012) The *Colletotrichum gloeosporioides* species complex. Studies in Mycology 73: 115–180. 23136459 10.3114/sim0011 PMC3458417

[17] Cannon PF, Buddie AG, Bridge PD (2008) The typification of *Colletotrichum gloeosporioides* . Mycotaxon 104: 189–204.

[18] Halsted B (1889) Some fungus diseases of the cranberry. New Jersey Agricultural College Experiment Station. 1–40.

[19] Shear CL (1907) Cranberry Diseases. In: USDA, editor: Government Printing Office.

[20] Stiles CM, Oudemans PV (1997) Cranberry fruit rot pathogens isolated from various cranberry plant tissues over two growing seasons. Phytopathology 87: S94.

[21] Zhu P (1999) Genetic diversity and systematics of *Colletotrichum* species on cranberry (*Vaccinium macrocarpon*) [Ph.D.]. New Brunswick: Rutgers University. 143 p.

[22] Olatinwo RO, Hanson EJ, Schilder AMC (2003) A First Assessment of the Cranberry Fruit Rot Complex in Michigan. Plant Disease 87: 550–556. 10.1094/PDIS.2003.87.5.550 30812957

[23] Polashock JJ, Caruso FL, Oudemans PV, McManus PS, Crouch JA (2009) The North American cranberry fruit rot fungal community: a systematic overview using morphological and phylogenetic affinities. Plant Pathology 58: 1116–1127.

[24] Ellis JB, Everhart BM (1894) New Species of Fungi from Various Localities Proceedings of the Academy of Natural Sciences of Philadelphia. 46: 322–386.

[25] Bills G, Polishook JD (1992) Recovery of endophytic fungi from *Chamaecyparis thyoides* . Sydowia 44: 1–12.

[26] Johnson DA, Carris LM, Rogers JD (1997) Morphological and molecular characterization of *Colletotrichum nymphaeae* and *C. nupharicola* sp. nov. on water-lilies (*Nymphaea* and *Nuphar*). Mycological Research 101: 641–649.

[27] Stiles CM, Oudemans PV (1999) Distribution of cranberry fruit-rotting fungi in New Jersey and evidence for nonspecific host resistance. Phytopathology 89: 218–225. 18944762 10.1094/PHYTO.1999.89.3.218

[28] Rodriguez-Saona C, Vorsa N, Singh AP, Johnson-Cicalese J, Szendrei Z, et al (2011) Tracing the history of plant traits under domestication in cranberries: potential consequences on anti-herbivore defences. Journal of Experimental Botany 62: 2633–2644. 21289080 10.1093/jxb/erq466

[29] Vorsa N, Polashock JJ (2005) Alteration of Anthocyanin Glycosylation in Cranberry Through Interspecific Hybridization. Journal of the American Society for Horticultural Science 130: 711–715.

[30] Vorsa N (1994) Breeding the American Cranberry. In: Roper TR, editor. Wisconsin State Cranberry Growers Association.

[31] Freeman S, Katan T, Shabi E (1996) Characterization of *Colletotrichum gloeosporioides* isolates from avocado and almond fruits with molecular and pathogenicity tests. Applied and Environmental Microbiology 62: 1014–1020. 8975596 10.1128/aem.62.3.1014-1020.1996 PMC167866

[32] Photita W, Taylor PWJ, Ford R, Hyde KD (2005) Morphological and molecular characterization of *Colletotrichum* species from herbaceous plants in Thailand. Fungal Diversity 18: 117–133.

[33] González E, Sutton TB, Correll JC (2006) Clarification of the etiology of *Glomerella* Leaf Spot and Bitter Rot of apple caused by *Colletotrichum* spp. based on morphology and genetic, molecular, and pathogenicity tests. Phytopathology 96: 982–992. 18944054 10.1094/PHYTO-96-0982

[34] Agostini JP, Timmer LW (1994) Population dynamics and survival of strains of *Colletotrichum gloeosporioides* on Citrus in Florida. Phytopathology 84: 420–425.

[35] Chakraborty S, Fernandes CD, Charchar MJdA, Thomas MR (2002) Pathogenic variation in *Colletotrichum gloeosporioides* infecting *Stylosanthes* spp. in a center of diversity in Brazil. Phytopathology 92: 553–562. 18943031 10.1094/PHYTO.2002.92.5.553

[36] Colon-Garay J, Rivera-Vargas L, McGovern RJ, Rodriquez RdP (2002) Hypovirulent isolates of *Colletotrichum gloeosporioides* induce resistance to anthracnose in detached mango fruits and seedlings. J Agric Univ Puerto Rico 84: 56–64.

[37] Kaufmann PJ, Weidemann GJ (1996) Isozyme analysis of *Colletotrichum gloeosporioides* from five host genera. Plant Disease 80: 1289–1293.

[38] MacKenzie SJ (2005) Population structure and pathogenicity of *Colletotrichum gloeosporioides* from strawberry and noncultivated hosts in Florida [Ph.D.]. United States – Florida: University of Florida.

[39] White TJ, Bruns T, Lee JS, Taylor JW (1990) Amplification and direct sequencing of fungal ribosomal RNA genes for phylogenetics. In: Innis MA, Gelfand DH, Sninsky JJ, White TJ, editors. PCR Protocols: A Guide to Methods and Applications. New York: Academic Press, Inc. 315–322.

[40] O’Donnell K, Cigelnik E (1997) Two divergent intragenomic rDNA ITS2 types within a monophyletic lineage of the fungus *Fusarium* are nonorthologous. Molecular Phylogenetics and Evolution 7: 103–116. 9007025 10.1006/mpev.1996.0376

[41] Katoh K, Kuma K-I, Toh H, Miyata T (2005) MAFFT version 5: improvement in accuracy of multiple sequence alignment. Nucleic Acids Research 33: 511–518. 15661851 10.1093/nar/gki198 PMC548345

[42] Katoh K, Toh H (2008) Recent developments in the MAFFT multiple sequence alignment program. Briefings in Bioinformatics 9: 286–298. 18372315 10.1093/bib/bbn013

[43] Goloboff PA, Farris JS, Nixon KC (2008) TNT, a free program for phylogenetic analysis. Cladistics 24: 774–786.

[44] Miller MA, Pfeifer W, Schwartz T (2010) Creating the CIPRES Science Gateway for inference of large phylogenetic trees; New Orleans, LA. 1–8.

[45] Stamatakis A (2006) RAxML-VI-HPC: Maximum Likelihood-based Phylogenetic Analyses with Thousands of Taxa and Mixed Models. Bioinformatics 22: 2688–2690. 16928733 10.1093/bioinformatics/btl446

[46] Stamatakis A, Hoover P, Rougemont J (2008) A Fast Bootstrapping Algorithm for the RAxML Web-Servers. Systematic Biology 57: 758–771. 18853362 10.1080/10635150802429642

[47] Huelsenbeck JP, Ronquist F (2001) MRBAYES: Bayesian inference of phylogeny. Bioinformatics 17: 754–755. 11524383 10.1093/bioinformatics/17.8.754

[48] Ronquist F, Huelsenbeck JP (2003) MRBAYES 3: bayesian phylogenetic inference under mixed models. Bioinformatics 19: 1572–1574. 12912839 10.1093/bioinformatics/btg180

[49] Nylander JAA (2004) MrModeltest v2. Program distributed by the author. Evolutionary Biology Centre, Uppsala University.

[50] Than PP, Shivas RG, Jeewon R, Pongsupasamit S, Marney TS, et al (2008) Epitypification and phylogeny of *Colletotrichum acutatum* J.H. Simmonds. Fungal Diversity 28: 97–108.

[51] Phoulivong S, Cai L, Chen H, McKenzie EHC, Abdelsalam K, et al (2010) *Colletotrichum gloeosporioides* is not a common pathogen on tropical fruits. Fungal Diversity 44: 33–43.

[52] Simmonds JH (1965) A study of the species of *Colletotrichum* causing ripe fruit rots in Queensland. Queensland Journal of Agricultural and Animal Science 25: 437–459.

[53] Sukumaran J, Holder MT (2010) DendroPy: A Python library for phylogenetic computing. Bioinformatics 26: 1569–1571. 20421198 10.1093/bioinformatics/btq228

[54] Rambaut A, Drummond AJ (2007) Tracer v.1.4. Beast website. Available: http://beast.bio.ed.ac.uk/Tracer. Accessed March 25, 2013.

[55] Ané C, Larget B, Baum DA, Smith SD, Rokas A (2007) Bayesian estimation of concordance among gene trees. Molecular Biology and Evolution 24: 412–426. 17095535 10.1093/molbev/msl170

[56] Larget BR, Kotha SK, Dewey CN, Ané C (2010) BUCKy: Gene tree/species tree reconciliation with Bayesian concordance analysis. Bioinformatics 26: 2910–2911. 20861028 10.1093/bioinformatics/btq539

[57] Phoulivong S, Cai L, Parinn N, Chen H, Abd-Elsalam KA, et al (2010) A new species of *Colletotrichum* from *Cordyline fruticosa* and *Eugenia javanica* causing anthracnose disease. Mycotaxon 114: 247–257.

[58] Prihastuti H, Cai L, Chen H, McKenzie EHC, Hyde KD (2009) Characterization of *Colletotrichum* species associated with coffee berries in northern Thailand. Fungal Diversity 39: 89–109.

[59] Silva DN, Talhinas P, Cai L, Manuel L, Gichuru EK, et al (2012) Host-jump drives rapid and recent ecological speciation of the emergent fungal pathogen *Colletotrichum kahawae* . Molecular Ecology 21: 2655–2670. 22519519 10.1111/j.1365-294X.2012.05557.x

[60] Dettman JR, Jacobson DJ, Taylor JW (2003) A multilocus genealogical approach to phylogenetic species recognition in the model eukaryote *Neurospora* . Evolution 57: 2703–2720. 14761051 10.1111/j.0014-3820.2003.tb01514.x

[61] Taylor JW, Jacobson DJ, Kroken S, Kasuga T, Geiser DM, et al (2000) Phylogenetic Species Recognition and Species Concepts in Fungi. Fungal Genetics and Biology 31: 21–32. 11118132 10.1006/fgbi.2000.1228

[62] Cummings MP, Neel MC, Shaw KL (2008) A genealogical approach to quantifying lineage divergence. Evolution 62: 2411–2422. 18564377 10.1111/j.1558-5646.2008.00442.x

[63] Bazinet AL, Myers DS, Khatavkar P (2009) genealogicalSorting v.0.91. Genealogical sorting index website. Available: http://www.genealogicalsorting.org/. Accessed March 31, 2013.

[64] R-Development-Core-Team (2011) R: A Language and Environment for Statistical Computing.

[65] Nirenberg H (1976) Untersuchungen über die morphologische und biologische Differenzierung in der Fusarium-Sektion Liseola. Mitteilungen aus der Biologischen Bundesanstalt für Land-und Forstwirtschaft 169: 1–117.

[66] Rasband WS (1997–2008) ImageJ. Bethesda, Maryland, USA: National Institutes of Health.

[67] Abramoff MD, Magelhaes PJ, Ram SJ (2004) Image Processing with ImageJ. Biophotonics International 11: 36–42.

[68] Seifert KA (2009) Progress towards DNA barcoding of fungi. Molecular Ecology Resources 9: 83–89. 10.1111/j.1755-0998.2009.02635.x 21564968

[69] Crouch JA, Clarke BB, Hillman BI (2009) What is the value of ITS sequence data in *Colletotrichum* systematics and species diagnosis? A case study using the falcate spored graminicolous *Colletotrichum* group. Mycologia 101: 648–656. 19750944 10.3852/08-231

[70] Shear CL (1907) New species of fungi. The Bulletin of the Torrey Botanical Club 34: 305–317.

[71] Wikee S, Cai L, Pairin N, McKenzie EHC, Su YY, et al (2011) *Colletotrichum* species from Jasmine (*Jasminum sambac*). Fungal Diversity 46: 171–182.

[72] Yang YL, Liu ZY, Cai L, Hyde KD (2012) New species and notes of *Colletotrichum* on daylilies (*Hemerocallis* spp.). Tropical Plant Pathology 37: 165–174.

[73] Yang YL, Liu ZY, Cai L, Hyde KD, Yu ZN, et al (2009) *Colletotrichum* anthracnose of Amaryllidaceae. Fungal Diversity 39: 123–146.

[74] Brasier CM (1995) Episodic selection as a force in fungal microevolution, with special reference to clonal speciation and hybrid introgression. Canadian Journal of Botany 73: S1213–S1221.

[75] Giraud T, Gladieux P, Gavrilets S (2010) Linking the emergence of fungal plant diseases with ecological speciation. Trends in Ecology and Evolution 25: 387–395. 20434790 10.1016/j.tree.2010.03.006 PMC2885483

[76] Gladieux P, Guerin F, Giraud T, Caffier V, Lemaire C, et al (2011) Emergence of fungal pathogens by ecological speciation: importance of the reduced viability of immigrants. Molecular Ecology 20: 4521–4532. 21967446 10.1111/j.1365-294X.2011.05288.x

[77] Crouch JA, Tredway LP, Clarke BP, Hillman BI (2009) Phylogenetic and population genetic divergence correspond with habitat for the pathogen *Colletotrichum cereale* and allied taxa across diverse grass communities. Molecular Ecology 18: 123–135. 19076279 10.1111/j.1365-294X.2008.04008.x

[78] Chilton SJP, Lucas GB, Edgerton CW (1945) Genetics of *Glomerella* III: Crosses with a conidial strain. American Journal of Botany 32: 549–554.

[79] Edgerton CW (1914) Plus and minus strains in the Genus *Glomerella* . American Journal of Botany 1: 244–254.

[80] Edgerton CW, Chilton SJP, Lucas GB (1945) Genetics of *Glomerella* II: Fertilization between strains. American Journal of Botany 32: 115–118.

[81] MacKenzie SJ, Seijo TE, Legard DE, Timmer LW, Peres NA (2007) Selection for pathogenicity to strawberry in populations of *Colletotrichum gloeosporioides* from native plants. Phytopathology 97: 1130–1140. 18944178 10.1094/PHYTO-97-9-1130

[82] Shear CL, Wood A (1907) Ascogenous forms of *Gloeosporium* and *Colletotrichum* . Botanical Gazette 43: 259–266.

[83] Wheeler HE, McGahen JW (1952) Genetics of *Glomerella* X: Genes affecting sexual reproduction. American Journal of Botany 39: 110–119.

