# Supplementary figures and images for: Habitat and Host Indicate Lineage Identity in Colletotrichum gloeosporioides s.l. from Wild and Agricultural Landscapes in North America
Source: PLoS One. 2013 May 6;8(5):e62394. doi: 10.1371/journal.pone.0062394 (PMC3646003; doi:10.1371/journal.pone.0062394)

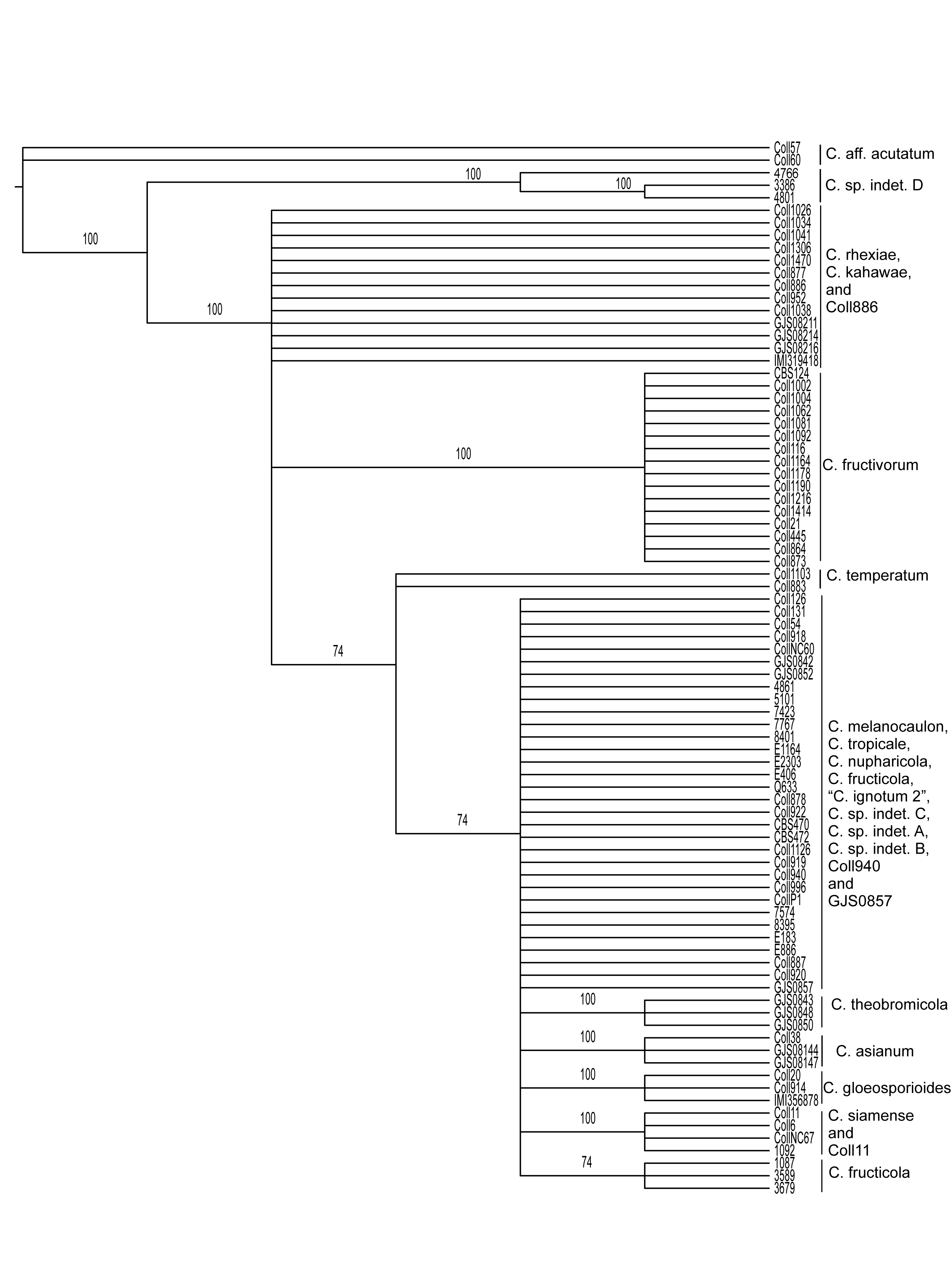

Supplement: Figure S1 — Nelsen consensus from maximum parsimony analysis of nrITS gene from D4G. Bootstrap support values shown above branches. (TIF) [file pone.0062394.s001.tif]

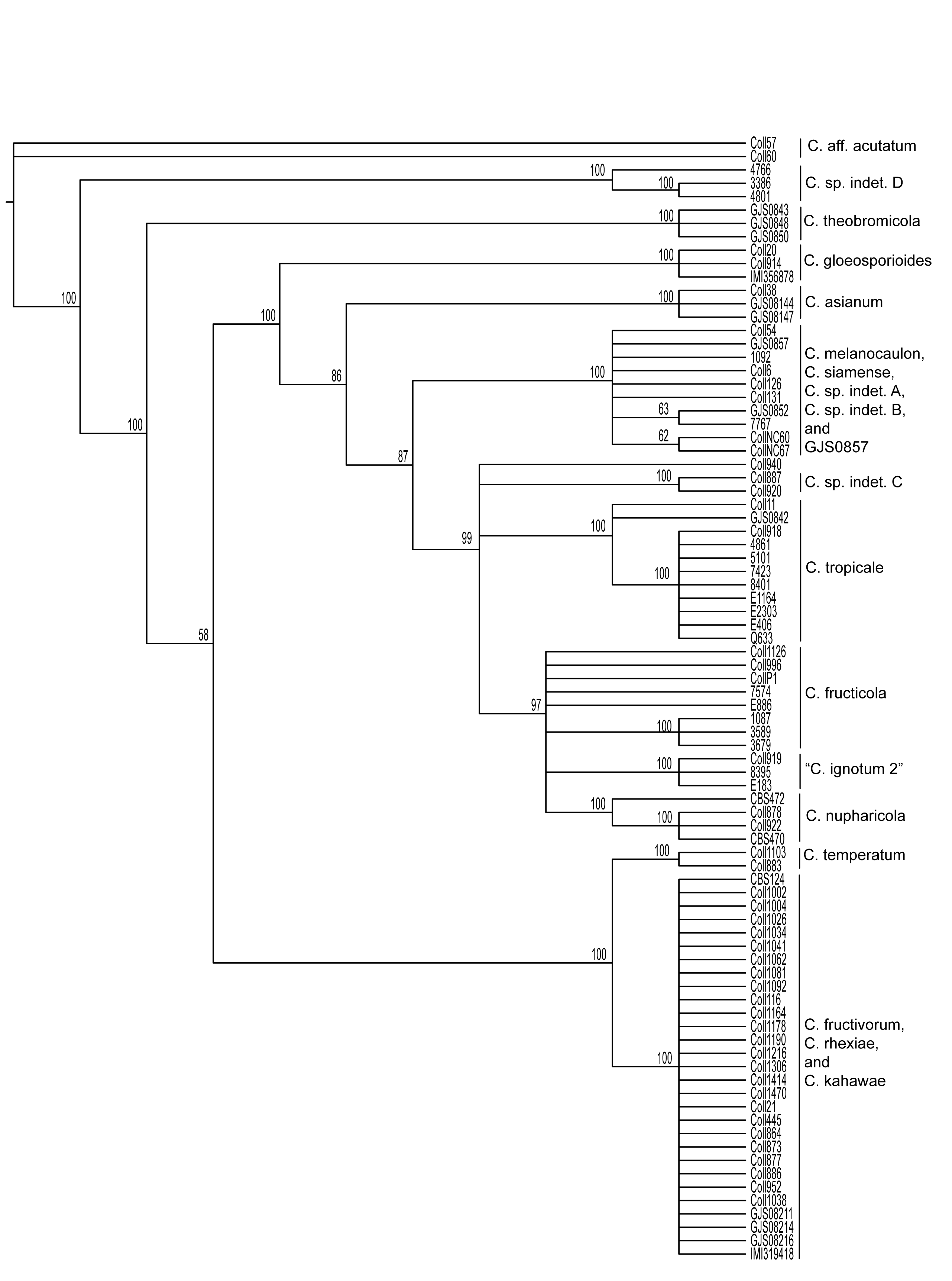

Supplement: Figure S2 — Nelsen consensus from maximum parsimony analysis of partial beta-tubulin gene from D4G. Bootstrap support values shown above branches. (TIF) [file pone.0062394.s002.tif]

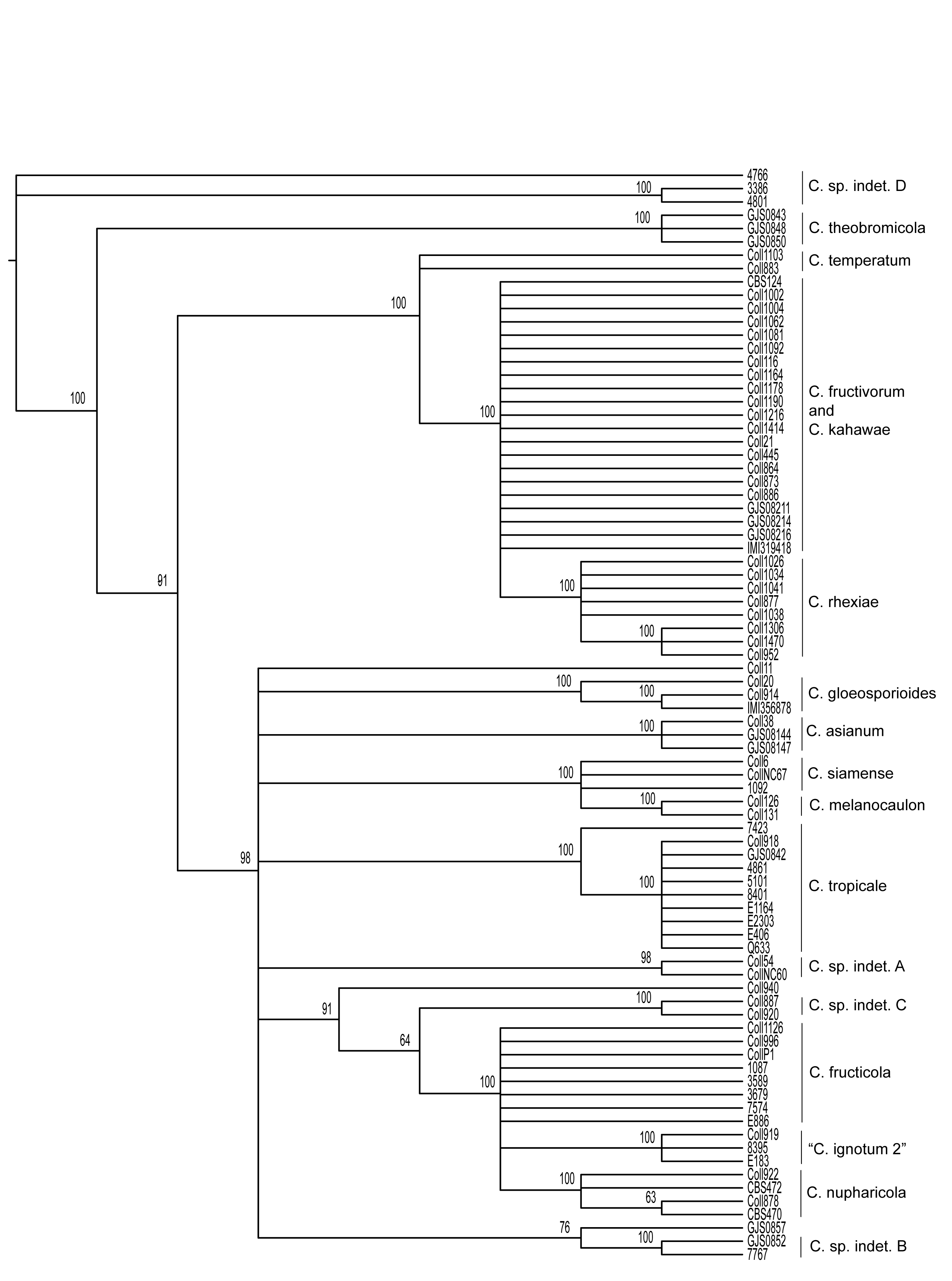

Supplement: Figure S3 — Nelsen consensus from maximum parsimony analysis of apn2 gene from D4G . Bootstrap support values shown above branches. (TIF) [file pone.0062394.s003.tif]

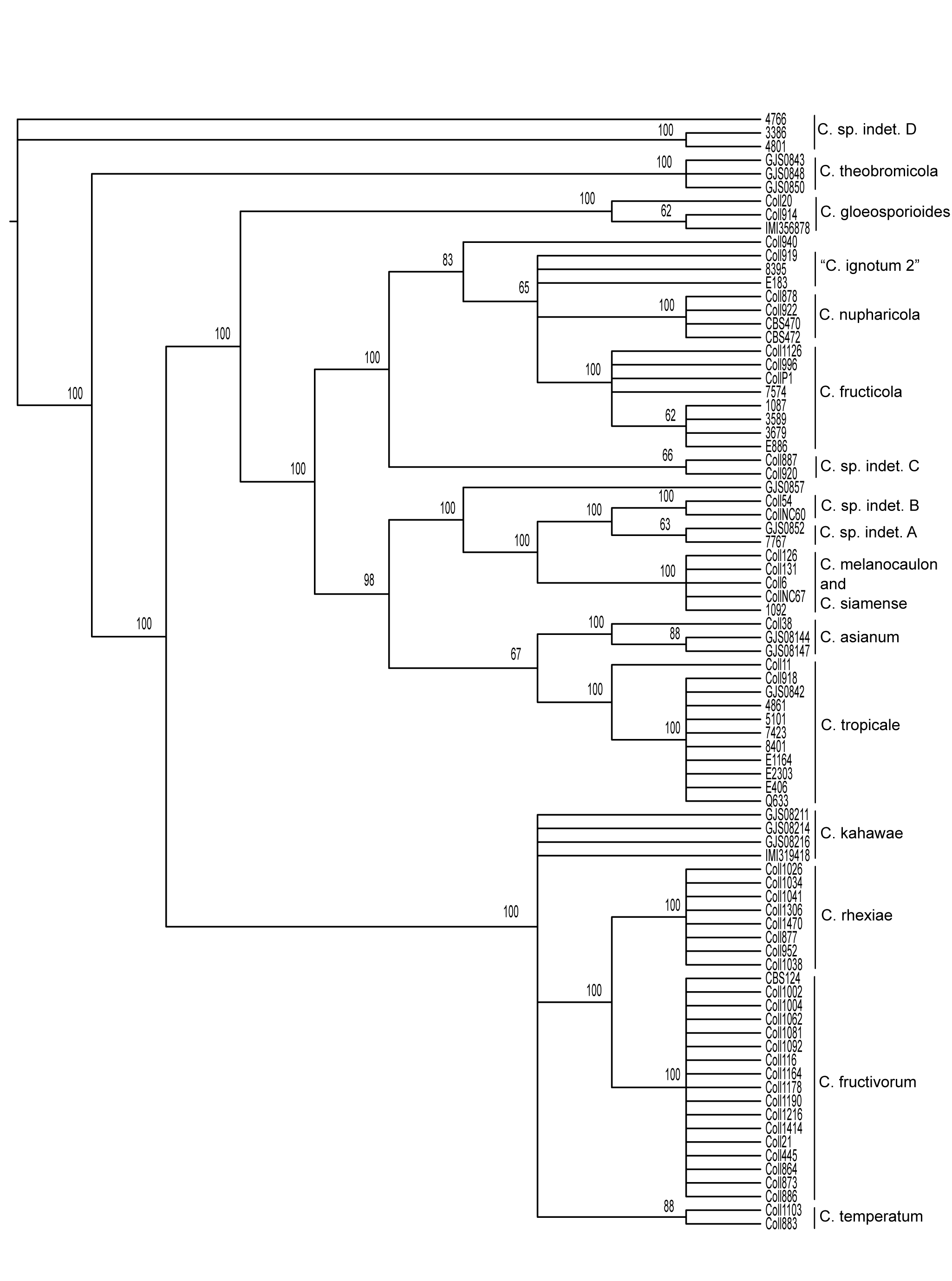

Supplement: Figure S4 — Nelsen consensus from maximum parsimony analysis of apn2/matIGS gene from D4G . Bootstrap support values shown above branches. (TIF) [file pone.0062394.s004.tif]

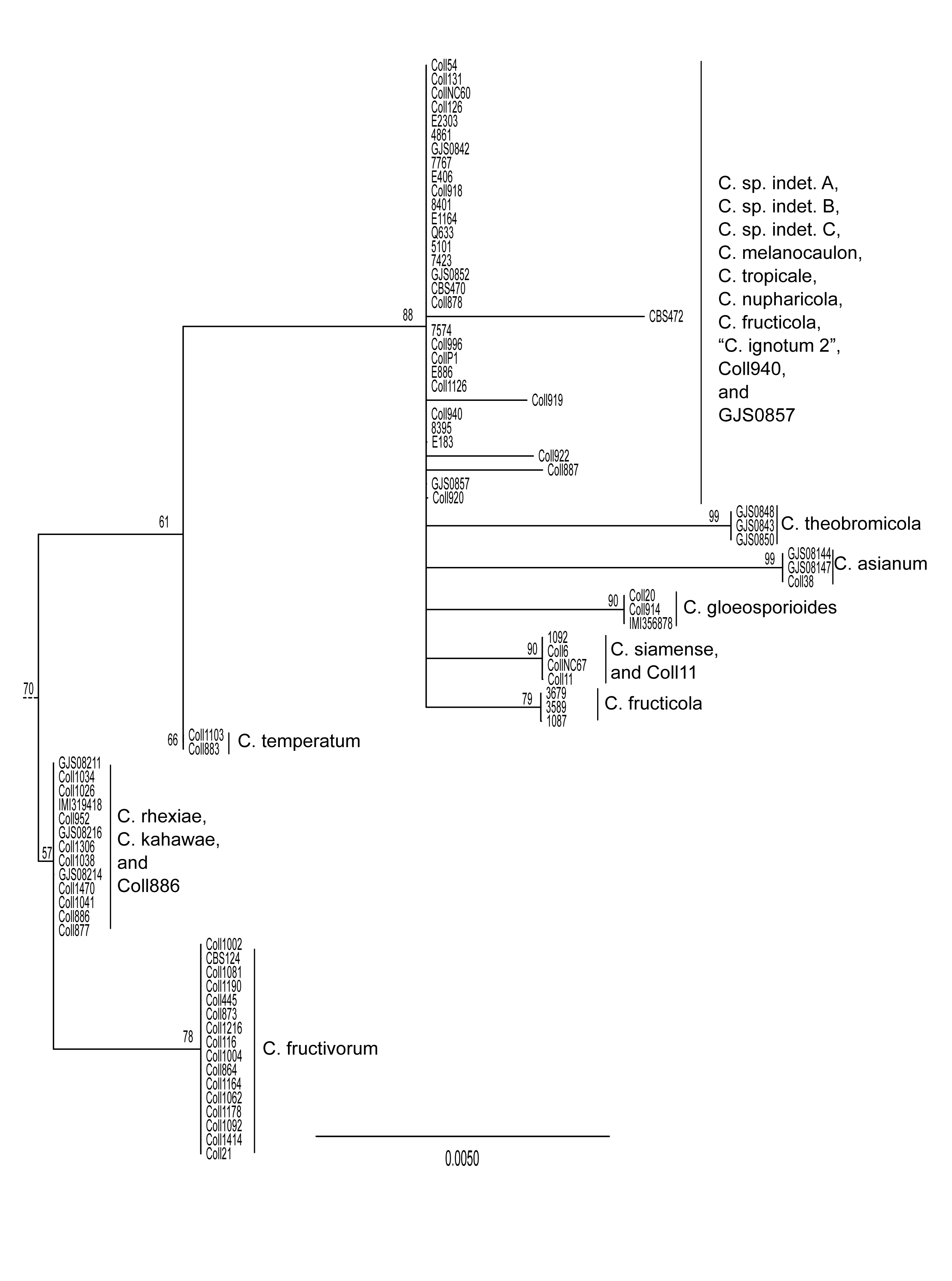

Supplement: Figure S5 — Maximum-likelihood majority-rule consensus tree of nrITS gene from D4G . Bootstrap support values shown above branches. Outgroups (C. aff. acutatum and strains 4766, 3386, and 4801) have been trimmed from the tree. Branch lengths represent the mean across samples. (TIF) [file pone.0062394.s005.tif]

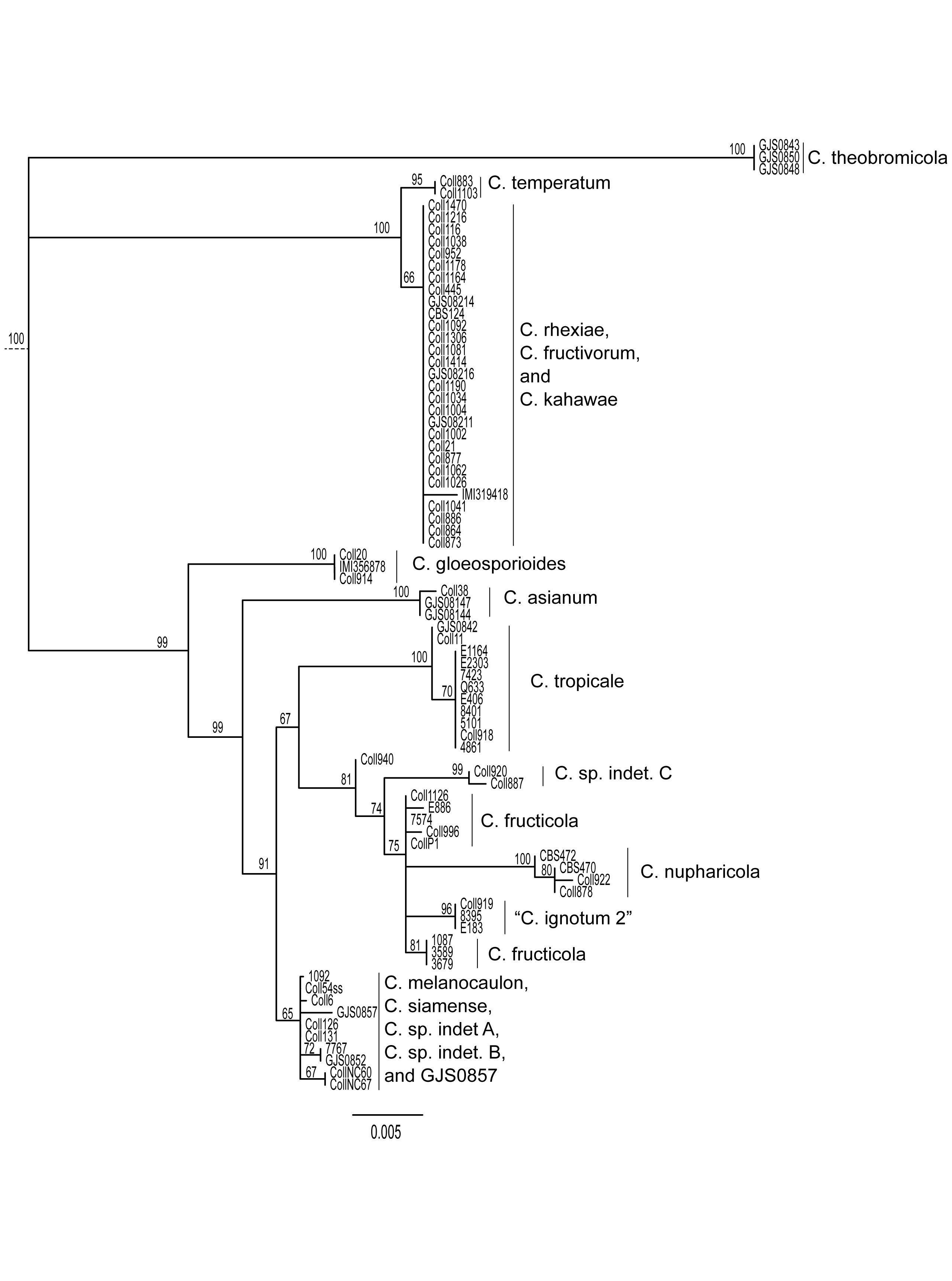

Supplement: Figure S6 — Maximum-likelihood majority-rule consensus tree of partial beta-tubulin gene from D4G . Bootstrap support values shown above branches. Outgroups (C. aff. acutatum and strains 4766, 3386, and 4801) have been trimmed from the tree. Branch lengths represent the mean across samples. (TIF) [file pone.0062394.s006.tif]

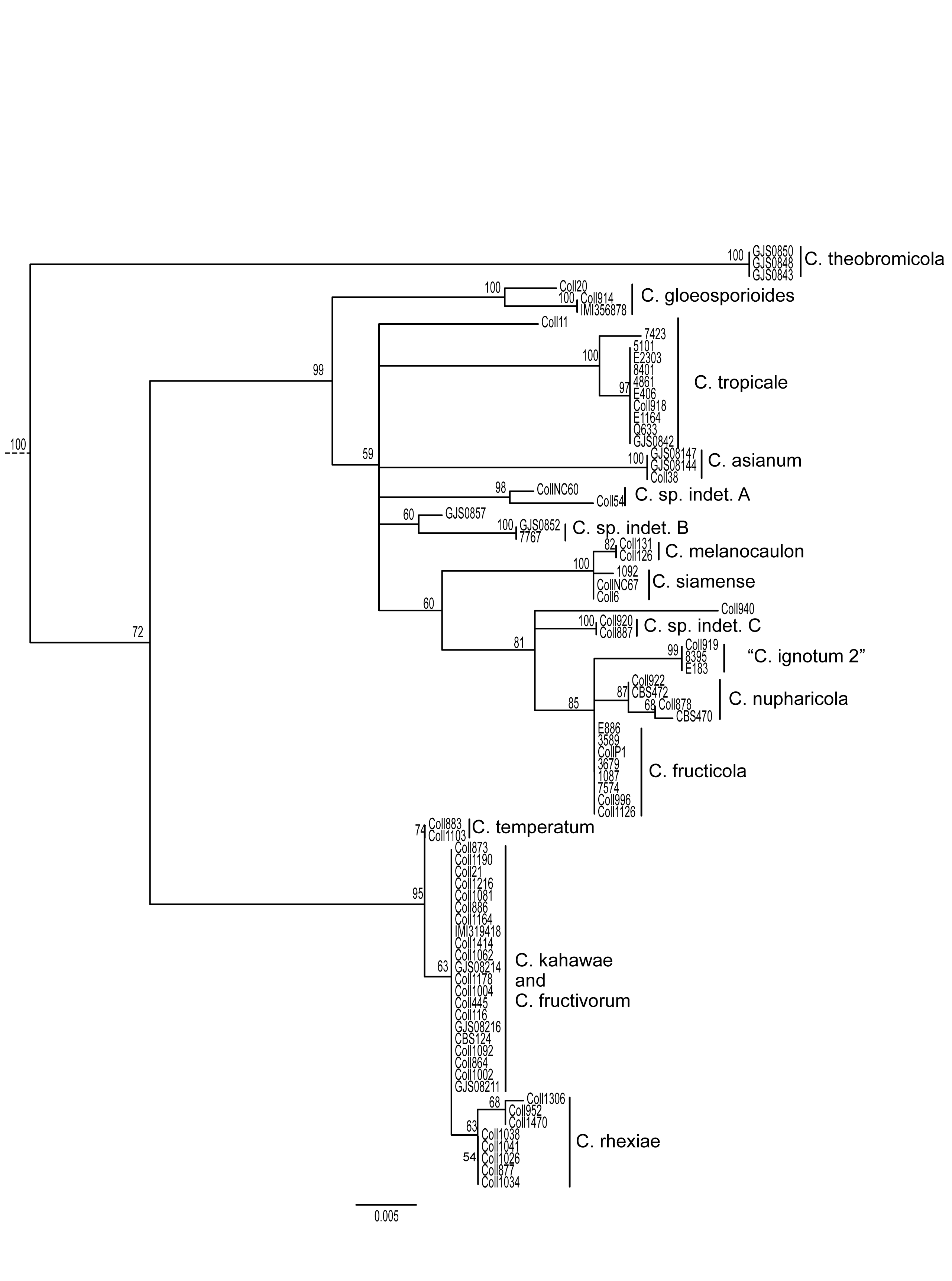

Supplement: Figure S7 — Maximum-likelihood majority-rule consensus tree of apn2 gene from D4G . Bootstrap support values shown above branches. Outgroups (C. aff. acutatum and strains 4766, 3386, and 4801) have been trimmed from the tree. Branch lengths represent the mean across samples. (TIF) [file pone.0062394.s007.tif]

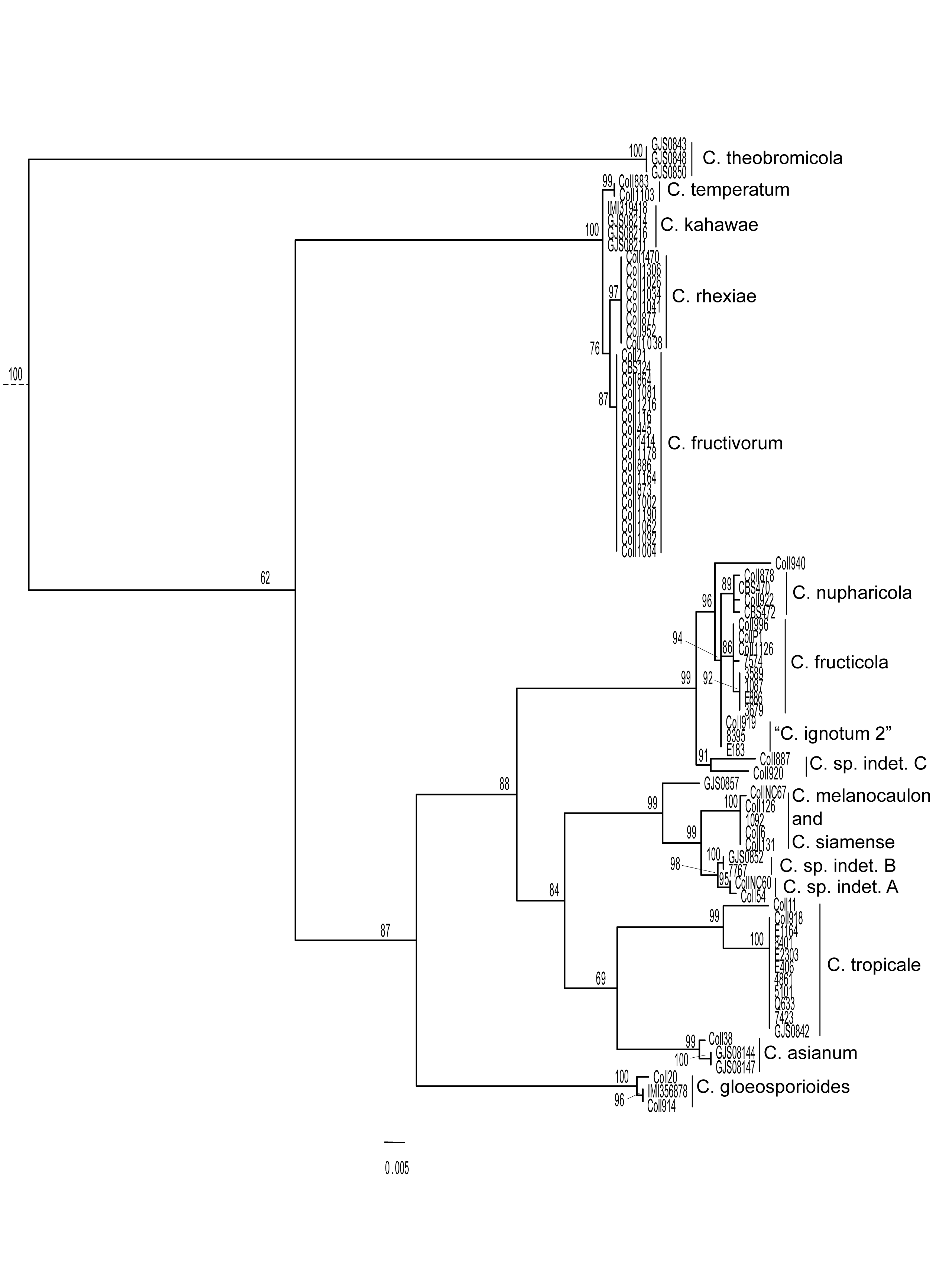

Supplement: Figure S8 — Maximum-likelihood majority-rule consensus tree of apn2/matIGS gene from D4G . Bootstrap support values shown above branches. Outgroups (C. aff. acutatum and strains 4766, 3386, and 4801) have been trimmed from the tree. Branch lengths represent the mean across samples. (TIF) [file pone.0062394.s008.tif]

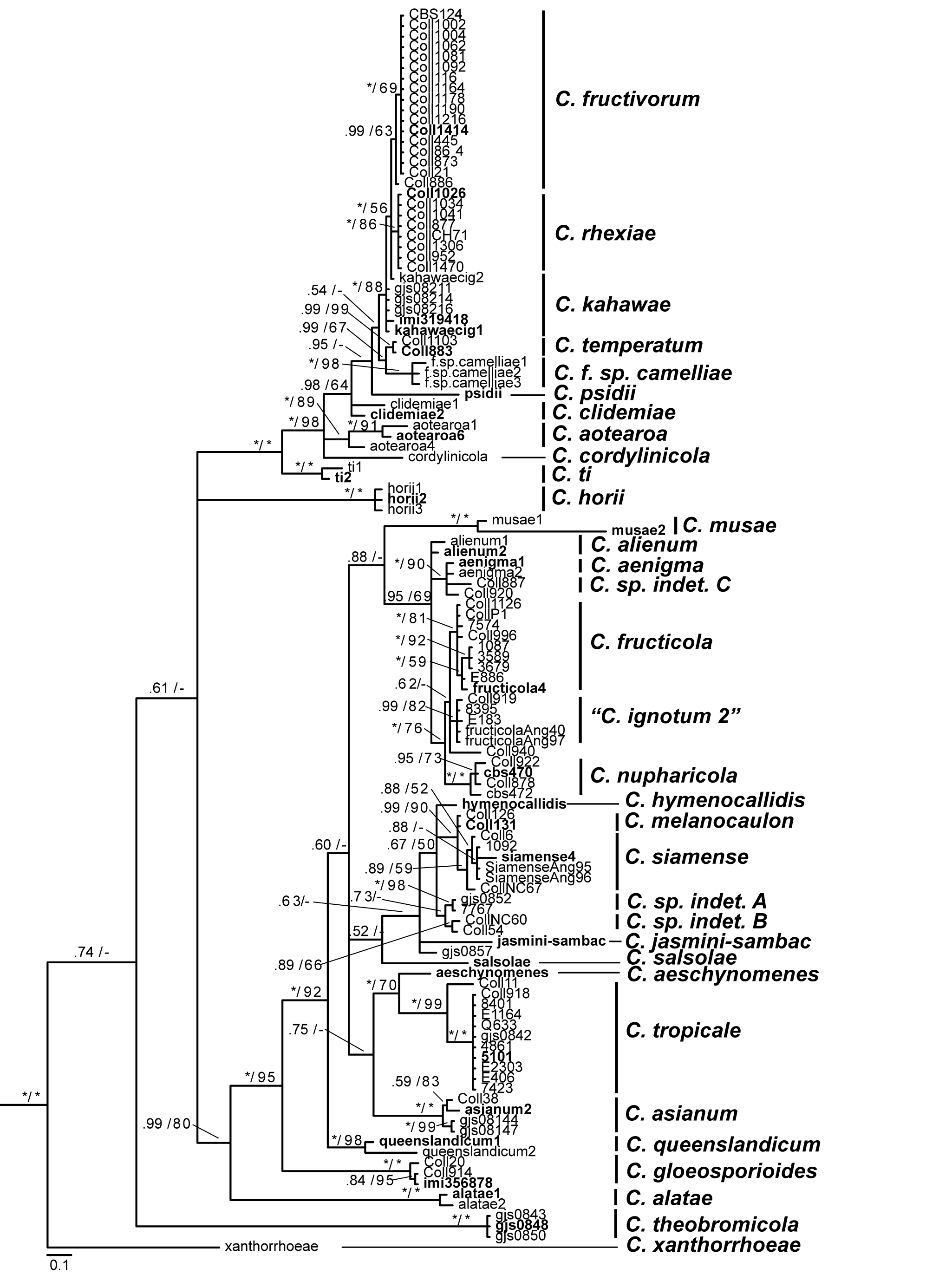

Supplement: Figure S9 — Bayesian majority-rule consensus tree with support values (PP/ML-BS) resulting from the combined analysis of D3G+. (TIF) [file pone.0062394.s009.tif]
