# Supplementary material for: Habitat and Host Indicate Lineage Identity in Colletotrichum gloeosporioides s.l. from Wild and Agricultural Landscapes in North America
Source: PLoS One. 2013 May 6;8(5):e62394. doi: 10.1371/journal.pone.0062394 (PMC3646003; doi:10.1371/journal.pone.0062394)
Supplement: Table S1 — GenBank accession numbers for sequence data generated in phylogenetic study of C. gloeosporioides s.l. (DOCX) [file pone.0062394.s010.docx]

| Strain | *nrITS* | *Tub2* | *APN2* | *APN2/MAT12IGS* | Species |
| --- | --- | --- | --- | --- | --- |
| CBS124 | JX145125 | JX145176 | JX145228 | JX145278 | *C. fructivorum* |
| CBS470 | JX145173 | JX145225 | JX145275 | JX145319 | *C. nupharicola* |
| CBS472 | JX145174 | JX145226 | JX145276 | JX145320 | *C. nupharicola* |
| Coll1002 | JX145126 | JX145177 | JX145229 | JX145279 | *C. fructivorum* |
| Coll1004 | JX145127 | JX145178 | JX145230 | JX145280 | *C. fructivorum* |
| Coll1026 | JX145128 | JX145179 | JX145231 | JX145290 | *C. rhexiae* |
| Coll1034 | JX145129 | JX145180 | JX145232 | JX145291 | *C. rhexiae* |
| Coll1038 | JX145168 | JX145220 | JX145270 | JX145295 | *C. rhexiae* |
| Coll1041 | JX145130 | JX145181 | JX145233 | JX145292 | *C. rhexiae* |
| Coll1062 | JX145131 | JX145182 | JX145234 | JX145281 | *C. fructivorum* |
| Coll1081 | JX145132 | JX145183 | JX145235 | JX145282 | *C. fructivorum* |
| Coll1092 | JX145133 | JX145184 | JX145236 | JX145283 | *C. fructivorum* |
| Coll11 | JX145134 | JX145185 | JX145237 | JX145306 | *C. tropicale* |
| Coll1103 | JX145135 | JX145186 | JX145238 | JX145297 | *C. temperatum* |
| Coll1126 | JX145136 | JX145187 | JX145239 | JX145315 | *C. fructicola* |
| Coll116 | JX145137 | JX145188 | JX145240 | JX145299 | *C. fructivorum* |
| Coll1164 | JX145138 | JX145189 | JX145241 | JX145284 | *C. fructivorum* |
| Coll1178 | JX145139 | JX145190 | JX145242 | JX145285 | *C. fructivorum* |
| Coll1190 | JX145140 | JX145191 | JX145243 | JX145286 | *C. fructivorum* |
| Coll1216 | JX145141 | JX145192 | JX145244 | JX145287 | *C. fructivorum* |
| Coll126 | JX145142 | JX145193 | JX145245 | JX145309 | *C. melanocaulon* |
| Coll1306 | JX145143 | JX145194 | JX145246 | JX145293 | *C. rhexiae* |
| Coll131 | JX145144 | JX145195 | JX145247 | JX145313 | *C. temperatum* |
| Coll1414 | JX145145 | JX145196 | JX145248 | JX145300 | *C. fructivorum* |
| Coll1470 | JX145146 | JX145197 | JX145249 | JX145304 | *C. rhexiae* |
| Coll20 | JX145148 | JX145199 | JX145251 | JX145326 | *C. gloeosporioides* |
| Coll21 | JX145149 | JX145200 | JX145252 | JX145301 | *C. fructivorum* |
| Coll38 | JX145150 | JX145201 | JX145253 | JX145308 | *C. asianum* |
| Coll445 | JX145151 | JX145202 | JX145254 | JX145288 | *C. fructivorum* |
| Coll54 | JX145152 | JX145203 | JX145255 | JX145311 | *C. sp. indet. B* |
| Coll57 | JX145124 | JX145204 | NS | NS | *C. aff. acutatum* |
| Coll6 | JX145153 | JX145205 | JX145256 | JX145314 | *C. siamense* |
| Coll60 | JX145154 | JX145206 | NS | NS | *C. aff. acutatum* |
| Coll864 | JX145155 | JX145207 | JX145257 | JX145305 | *C. fructivorum* |
| Coll873 | JX145156 | JX145208 | JX145258 | JX145289 | *C. fructivorum* |
| Coll877 | JX145157 | JX145209 | JX145259 | JX145302 | *C. rhexiae* |
| Coll878 | JX145158 | JX145210 | JX145260 | JX145323 | *C. nupharicola* |
| Coll883 | JX145159 | JX145211 | JX145261 | JX145298 | *C. temperatum* |
| Coll886 | JX145160 | JX145212 | JX145262 | JX145303 | *C. fructivorum* |
| Coll887 | JX145147 | JX145198 | JX145250 | JX145321 | *C. sp. indet. C* |
| Coll914 | JX145161 | JX145213 | JX145263 | JX145327 | *C. gloeosporioides* |
| Coll918 | JX145162 | JX145214 | JX145264 | JX145307 | *C. tropicale* |
| Coll919 | JX145163 | JX145215 | JX145265 | JX145317 | *“C. ignotum 2”* |
| Coll920 | JX145164 | JX145216 | JX145266 | JX145322 | *C. sp. indet. C* |
| Coll922 | JX145172 | JX145224 | JX145274 | JX145318 | *C. nupharicola* |
| Coll940 | JX145165 | JX145217 | JX145267 | JX145325 | ND |
| Coll952 | JX145166 | JX145218 | JX145268 | JX145294 | *C. rhexiae* |
| Coll996 | JX145167 | JX145219 | JX145269 | JX145324 | *C. fructicola* |
| CollNC60 | JX145169 | JX145221 | JX145271 | JX145312 | *C. sp. indet. B* |
| CollNC67 | JX145170 | JX145222 | JX145272 | JX145310 | *C. siamense* |
| CollP1 | JX145171 | JX145223 | JX145273 | JX145316 | *C. fructicola* |
| IMI319418 | JX145175 | JX145227 | JX145277 | JX145296 | *C. kahawae* |
| GJS08211 | KC735140 | KC735137 | KC735131 | KC735134 | *C. kahawae* |
| GJS08214 | KC735141 | KC735138 | KC735132 | KC735135 | *C. kahawae* |
| GJS08216 | KC735142 | KC735139 | KC735133 | KC735136 | *C. kahawae* |

NS – not sequenced

ND – species designation not made
