# Supplementary material for: Habitat and Host Indicate Lineage Identity in Colletotrichum gloeosporioides s.l. from Wild and Agricultural Landscapes in North America
Source: PLoS One. 2013 May 6;8(5):e62394. doi: 10.1371/journal.pone.0062394 (PMC3646003; doi:10.1371/journal.pone.0062394)
Supplement: Text S1 — Discussion of the inferences drawn from the analysis of D3G+. (DOCX) [file pone.0062394.s012.docx]

Supplemental Text:

To integrate the species described in this study with those in the species complex described since the epitypification of *C. gloeosporioides*, nrITS and partial *btub* sequences from ex-type strains were added to the data analyzed for Figure 3 to include all twenty-two species (plus one subspecies and a *forma specialis*) recently circumscribed within the species complex [[16](#_ENREF_16)]. We also included sequence data from ex-type strains of *C. jasmini-sambac* and *C. hymenocallidis*, two species considered synonyms of *C. siamense* by Weir et al. [[16](#_ENREF_16)]. The consensus tree resulting from the Bayesian analysis (Figure S9) supports the previous analyses in recovering two principal lineages within the species complex. Despite lower support values in both Bayesian and maximum likelihood bootstrap analyses, the tree in Figure S9 is not in conflict with the recognition of three new species and *C. rhexiae* appears to be distinct from recently described species. This analysis also highlights the fact that placing *C. jasmini-sambac* and *C. hymenocallidis* in synonymy with *C. siamense*, as in Weir et al. [[16](#_ENREF_16)] is, on this evidence, in error. Resolving relationships among species in this part of the species complex will be greatly assisted by generating sequence data for all markers that have been developed to date. However, it is likely that additional marker development efforts will be needed, as noted by Weir et al. [[16](#_ENREF_16)].
